# Uncovering Trait Associations Resulting in Maximal Seed Yield in Winter and Spring Oilseed Rape

**DOI:** 10.3389/fpls.2021.697576

**Published:** 2021-09-06

**Authors:** Laura Siles, Kirsty L. Hassall, Cristina Sanchis Gritsch, Peter J. Eastmond, Smita Kurup

**Affiliations:** ^1^Department of Plant Sciences, Rothamsted Research, Harpenden, United Kingdom; ^2^Department of Computational and Analytical Sciences, Rothamsted Research, Harpenden, United Kingdom

**Keywords:** *Brassica napus*, oilseed rape, ovule number, plant architecture, seed yield, seed number, seed size, trade-off

## Abstract

Seed yield is a complex trait for many crop species including oilseed rape (OSR) (*Brassica napus*), the second most important oilseed crop worldwide. Studies have focused on the contribution of distinct factors in seed yield such as environmental cues, agronomical practices, growth conditions, or specific phenotypic traits at the whole plant level, such as number of pods in a plant. However, how female reproductive traits contribute to whole plant level traits, and hence to seed yield, has been largely ignored. Here, we describe the combined contribution of 33 phenotypic traits within a *B. napus* diversity set population and their trade-offs at the whole plant and organ level, along with their interaction with plant level traits. Our results revealed that both Winter OSR (WOSR) and Spring OSR (SOSR); the two more economically important OSR groups in terms of oil production; share a common dominant reproductive strategy for seed yield. In this strategy, the main inflorescence is the principal source of seed yield, producing a good number of ovules, a large number of long pods with a concomitantly high number of seeds per pod. Moreover, we observed that WOSR opted for additional reproductive strategies than SOSR, presenting more plasticity to maximise seed yield. Overall, we conclude that OSR adopts a key strategy to ensure maximal seed yield and propose an ideal ideotype highlighting crucial phenotypic traits that could be potential targets for breeding.

## Introduction

Improving crop production, particularly seed yield, is vital to ensure food availability for an increasing population in the world. This challenge needs to be met in the face of climate change and reduced availability of arable land. Improving seed yield is a major goal for crop breeding programs for several crop species. *Brassica napus*, also known as rapeseed or oilseed rape (OSR), is the second most important oilseed crop globally (Food and Agriculture Organization of the United Nations, 2019) accounting for 20% of the world’s total oil production (Hu et al., 2017a). It is also a crucial source of high-quality protein for livestock and biofuel production (Raboanatahiry et al., 2018). Therefore, increasing its yield is vital to meet the high demands of oil and animal feed worldwide.

Seed yield in OSR is a complex trait affected by several factors such as environmental cues, agronomical practices, and growth conditions that influence source/sink capacity and resource allocation (Diepenbrock, 2000; Berry and Spink, 2006; Nesi et al., 2008; Hu et al., 2017a; Assefa et al., 2018). Studies have focused on the effect of temperature during plant development and growth (Weymann et al., 2015; Brown et al., 2019), plant density and row spacing (Kuai et al., 2015; Ren et al., 2017), nutrient requirements (Stahl et al., 2019), plant and canopy architecture (Bennett et al., 2012; Pinet et al., 2015), pod length (Li et al., 2019) as well as flowering time and petal morphogenesis (Schiessl et al., 2015; Kirkegaard et al., 2016; Raman et al., 2016; Yu et al., 2016) to understand and improve yield in *B. napus*. Studies on the relationships between seed yield components and seed yield have focused on a limited number of phenotypic traits, such as number of pods per plant, seed number per pod (SNPP), pod length, and number of branches per plant (Habekotté, 1997; Özer et al., 1999; Naazar et al., 2003; Badaran et al., 2007; Tunçtürk and Çiçti, 2007; Başalma, 2008; Sabaghnia et al., 2010; Chen et al., 2014; Ul-Hasan et al., 2014; Moradi et al., 2017; Ahmadzadeh et al., 2019; Tariq et al., 2020). However, only Sabaghnia et al. (2010) has focused on 20 phenotypic traits in 49 *B. napus* genotypes. Since plant development is complex, any study on seed yield should address the interplay of the various developmental traits and their combined effect.

Seed number per pod, pod number, and seed weight are considered the most significant components of yield in OSR (Yang et al., 2017), and studies have shed light on the genetic regulation of these traits and their role in seed yield (Li et al., 2015, 2019; Yang et al., 2016, 2017; Dong et al., 2018; Zhu et al., 2020). Specifically, SNPP shows a large variation within germplasm resources, from 5 to 35 seeds per pod (Chen et al., 2013). SNPP is determined by the number of ovules per ovary, the proportion of fertile ovules, the number of ovules fertilised, and the number of fertilised ovules that develop into seeds (Yang et al., 2016, 2017). However, the natural variation of SNPP and the regulation between ovule number and SNPP in different *B. napus* genotypes with different genetic backgrounds are poorly known, having been explored, so far, only in a limited capacity (Yang et al., 2017). Similarly, there is limited knowledge of the effect, if any, of female reproductive traits, such as ovule number and size and style, ovary, and gynoecia length on seed yield (Wang et al., 2011; Jiao et al., 2021).

Here, we present a comprehensive study on the contribution of 33 phenotypic traits and their trade-offs on seed yield, including traits at the whole plant level down to the less studied female reproductive traits. This study was performed within a *B. napus* diversity set population formed of 96 genotypes, classified in 4 OSR groups, subjected to the same vernalisation treatment. We analysed the relationships between the phenotypic traits by principal component analysis (PCA) at the whole population level, performing a principal component regression to relate them to seed yield. Subsequently, a partial least squares (PLS) analysis for Winter OSR (WOSR) and Spring OSR (SOSR), the two more economically important groups of OSR in terms of oil production, was performed. PCA and PLS are useful tools for plant breeding purposes as they allow us to identify combination of traits to explain the maximal variation in the data that can relate to seed yield. These analyses are more powerful than correlations, for example, which just estimate the simple linear relationship between two traits. PCA and PLS enable us to study several traits, and more importantly, the effect of their combination in relation to seed yield. PCA and PLS have been used to determine factors affecting yield in other crops, such as sweet potato (Rukundo et al., 2015), wheat (Hansen et al., 2002; Baranwal et al., 2013; Beheshtizadeh et al., 2013; Hu et al., 2017b; Devesh et al., 2019), and rice (Kumar et al., 2013; Pathak et al., 2018; Li, 2020; Guo et al., 2021).

The overall aims of this paper are to study developmental traits influencing seed yield in different OSR groups in a diversity set population and to elucidate the interrelations of these seed yield components. Furthermore, we wanted to identify reproductive strategies that influence seed yield, with a focus on WOSR and SOSR. We unravel the trade-offs between the measured traits at the whole plant level (macrotraits) and in addition, between female reproductive traits (microtraits) and their association to seed production. Finally, we aim to identify the best predictors of seed yield in WOSR and SOSR.

## Materials and Methods

### Plant Material and Growth Conditions

The *B. napus* diversity set population consisted of 96 genotypes that included WOSR, SOSR, Semiwinter OSR, swede, kales, unspecified, and Spring and Winter fodder genotypes (Harper et al., 2012; Havlickova et al., 2018). The population was classified in four OSR groups, including WOSR (42 lines), SOSR (22 lines), Semiwinter OSR (8 lines), and Others (24 lines which included swede, kale, unspecified, and fodder genotypes, Supplementary Table 1). The seeds were germinated in P24 trays with John Innes Cereal Mix as described in Siles et al. (2020), with one plant per pot. When the plants presented four true leaves, they were transferred to a vernalisation room with an 8 h photoperiod at 4°C day/night for 8 weeks. One plant per pot was re-potted in 2 L pots with John Innes Cereal Mix and were allocated in two glasshouse compartments in long-day conditions (16 h photoperiod) at 18°C day/15° night (600w SON-T, high pressure sodium lighting) at a density of 12 pots per m^2^. Plants were distributed in ebb-and-flow benches that were flooded twice a day for approximately 25 min, after which the water was drained to a reservoir. Once the plants started to mature, watering was reduced to once a day, decreasing the time of watering gradually until turning the water off completely. Perforated plastic bags (380 mm × 900 mm, WR Wright & Sons Ltd., Liverpool, United Kingdom) were used to enclose inflorescences to prevent cross-pollination from neighbouring plants once the plants started to bolt.

### Phenotyping

A total of 33 traits and seed yield were measured for the entire diversity set population, performing a total of 14,976 measurements. Seed yield and a further 26 phenotypic traits, measured on all 5 biological replicates of each genotype, were classified as macrotraits as they could be measured at the whole plant level. The other seven phenotypic traits were classified as microtraits, as these required some level of dissection prior to being measured and were performed on three biological replicates for each genotype. The combination of macrotraits and microtraits was classified as alltraits. A list of the names, units and abbreviations used for the 33 measured phenotypic traits and seed yield can be found in Table 1.

**TABLE 1:** List of macrotrait (*n* = 5) and microtrait (*n* = 3) names and abbreviations measured in the diversity set population.

Macrotraits		Microtraits	
	
Trait name	Abbreviation	Trait name	Abbreviation
Plant height (cm)	PH	Ovule number	ON
Number of flowering inflorescences	NI	Ovule area (mm^2^)	OA
Number of secondary inflorescences	NI-1	Ovary length (mm)	OL
Time to flowering (days)	TF	Gynoecia length (mm)	GL
Number of flowers on the whole plant	FN	Style length (mm)	SL
Number of pods on the main inflorescence	PN_*M*_	Beak length (cm)	BL
Number of pods on a secondary inflorescence	PN_1s_	Ovule area coefficient of variation (%)	OAcvar
Number of pods on secondary inflorescences	PN_*s*_		
Number of pods on the whole plant	PN		
Pod abortion on the main inflorescence (%)	PA_*M*_		
Pod abortion on a secondary inflorescence (%)	PA_1s_		
Pod abortion in secondary inflorescences (%)	PA_*s*_		
Pod abortion in the whole plant (%)	PA		
Time to maturity (days)	TM		
Pod length from 10 pods from the main inflorescence (cm)	PL_*M*_		
Valve length from 10 pods from the main inflorescence (cm)	VL_*M*_		
Seed number/pod from 10 pods from the main inflorescence	SNPP_*M*_		
Seed area from 10 pods from the main inflorescence (mm^2^)	SA_*M*_		
Seed compactness from 10 pods from the main inflorescence	SC_*M*_		
Seed weight from 10 pods from the main inflorescence (g)	SW_*M*_		
Seed area from the whole plant (mm^2^)	SA		
Seed compactness from the whole plant	SC		
Seed area coefficient of variation from whole plant (%)	SAcvar		
Thousand grain weight (g)	TGW		
Estimated total seed number from the whole plant (by TGW)	SN		
Seed oil content from the whole plant (%)	OC		
Seed weight from the whole plant (seed yield, g)	SY		

#### Macrotrait Phenotyping

Plants were monitored daily visually, and time to flowering was recorded when the first flower opened. Once the plants were dry, time to maturity, plant height, number of secondary inflorescences, number of pods and percentage of pod abortion in the main inflorescence were manually recorded.

Based on two best representative secondary inflorescences, the number of pods and the percentage of aborted pods for a single secondary inflorescence were determined. Moreover, we estimated the number of pods and percentage of aborted pods for all secondary inflorescences. The number of successful flowers on the whole plant was estimated by the number of developed and aborted pods on the whole plant.

Ten representative pods per plant from the main inflorescence between the 9th and the 19th pod were imaged (NIKON D5300 with NIKKOR 50 mm f1.8 prime lens, Minato, Tokyo, Japan). Subsequently, each pod was opened to remove the seeds, which were placed in individual petri dishes in order, and imaged. Pod and valve length were measured using SmartRoot tool in FIJI (Schindelin et al., 2012) and their average was calculated for each plant. The number of seeds per pod (SNPP_*M*_) was counted using Cell counter tool in FIJI, and its average was calculated for each plant. Seed area and compactness (a measure of the circularity of the seed) of seeds from 10 pods from the main inflorescence as well as from the whole plant were recorded (Videometer, Videometer A/S, Herlev, Denmark). For the latter measurement, three technical replicates for each plant were measured, and seed area and compactness were averaged for each plant.

Seed oil content was measured by time-domain nuclear-magnetic resonance (TD-NMR, Bruker minispec mq-20 NMR, Bruker, MA, United States) for each plant [standardised by seed moisture content at 9% (Van Erp et al., 2014)]. Thousand grain weight (TGW) was calculated from a sample of 200 seeds from each plant, and the number of total seeds per plant was estimated by TGW. Finally, seed weight from 10 pods from the main inflorescence as well as from the whole plant (seed yield) were obtained. Seed yield was measured as the total weight of all seeds produced by a single plant.

#### Microtrait Phenotyping

A total of three buds per plant at stages 12–13 (Sanders et al., 1999) were collected 24 h prior to anthesis (pre-fertilisation stage) between buds 6 and 20 from the main inflorescence for three biological reps per genotype. Sepals, petals and anthers were removed, obtaining three gynoecia per plant placed in a glass vial with 4% paraformaldehyde in 0.01 M Phosphate Buffer Saline and stored at 4°C until further processing. For each plant, an image of the three gynoecia using a stereo microscope (Leica M-205, Leica microsystems) was captured. Then, the ovules were extracted from the ovaries and imaged. Ovary, style, and gynoecia length as well as ovule area and number were measured from these images using FIJI. For each plant, the average of three technical reps was measured. Beak length (length of pod tip at the opposite end from the pedicel) from 10 mature pods from the main inflorescence was measured using SmartRoot tool in FIJI, and its average was calculated for each plant.

#### Ovule, Seed Area, and Seed Number per Pod Coefficient of Variation

Each biological replicate contained between 70 and 120 ovule measurements taken from three gynoecia (around 30–40 measurements per gynoecia). Consequently, the percentage coefficient of variation of ovule area was calculated for each plant,

$$\%CV=\frac{s\,d}{m\,e\,a\,n}\times 100$$

where SD is the standard deviation of all ovule measurements (within a single plant) and mean is the average ovule area. Similarly, the percentage coefficient of variation of seed area was calculated per plant, where between 300 and 1200 measurements were available per plant, and the coefficient of variation for SNPP was calculated from 10 pods per plant with a small number of exceptions (one plant had six pods and two plants had nine pods).

### Statistical Analyses

#### Statistical Design

Ninety-six genotypes with five biological replicates were arranged in two glasshouses. Each glasshouse contained all 96 genotypes arranged in a 20 × 12 non-resolvable row–column design. All genotypes were replicated either two or three times per glasshouse to give a total of five replicates across both glasshouses. The design was generated in CycDesigN (CycDesigN 6.0, VSN International Ltd., Hertfordshire, United Kingdom).

#### Univariate Analysis

Each trait was analysed using a linear mixed model. The block structure was defined by glasshouse/(row × column), and the main effect of glasshouse was fitted as a fixed effect. Glasshouse.row and glasshouse.column were both fitted as random effects. The treatment term accounting for differences between genotypes was fitted as a fixed effect, with statistical significance assessed by the Kenward–Roger approximate *F*-tests (Kenward and Roger, 1997) after having fitted the main effect of glasshouse. Further refinement of the random model was done on a trait-by-trait basis, and where necessary, variables were transformed to satisfy homogeneity of variance (Supplementary Table 2).

The three traits related to pod abortion percentages (main inflorescence, secondary inflorescences and whole plant) were analysed on the logit scale with the associated number of pods (on main inflorescence, on secondary inflorescences and on whole plant, respectively) included as a weight. For the 23 macrotraits (all 26 excluding the three traits related to pod abortion percentages) independent AR(1)–AR(1) correlated error structures were imposed on the rows and columns of each glasshouse.

#### Principal Component Analysis

Principal component analysis was performed on (1) the set of 26 macrotraits (PCA_macro_) and (2) the set of 33 traits (PCA_alltraits_) using the NIPALS algorithm implemented in the mixOmics package of R (Rohart et al., 2017) and run using the correlation matrix. Input variables were adjusted for glasshouse and position within glasshouse as per the univariate analysis and kept on the transformed scale where applicable. For PCA_macro_, 12 principal components (PCs) were retained, explaining 95.46% of the variation in the data. For PCA_alltraits_, 16 PCs were retained, explaining 95.96% of the variation in the data.

#### Principal Component Regression

To understand which traits were associated with the observed yield differential (the variation in seed yield), a principal component regression analysis was carried out. This consisted of two parts (1) for the macrotraits only, using PCA_macro_ and (2) for alltraits subsetting the data to three replicates per genotype using PCA_alltraits_. For the macrotraits, a baseline model for seed yield was defined as per the above univariate analysis. Specifically, a linear mixed model with random model defined by glasshouse(row × column) and fixed model defined by glasshouse + genotype. Two additional auto-correlated error terms were fitted across the rows and across the columns within each glasshouse to further account for the spatial dependence. The principal component regression models kept the same random structure with correlated error terms, but with fixed model consisting of glasshouse + OSRgroup × (PC1 + PC2 + … + PC12). Significance of individual terms was assessed by the marginal Kenward–Roger *F*-statistic (Kenward and Roger, 1997). An approximate percentage variance each model accounted for was calculated according to,

$$\%var_{a\,p\,p\,r\,o\,x}=100\times \frac{v\,a\,r_{n\,u\,l\,l}-v\,a\,r_{x}}{v\,a\,r_{n\,u\,l\,l}}$$

where *var*_*null*_ is the sum of the variance components under a model with no fixed effects beyond glasshouse and *var_x_* is the sum of the variance components under a model with a defined fixed model (Welham et al., 2015). For the combined set of macro and microtraits, restricted to the three replicates per genotype, the principal component regression modelling was performed in the same way as above, with the exception that no autocorrelated spatial error terms were included in the mixed models and a maximum of 16 PCs were allowed. Analysis of the contribution of each PC to seed yield was compared across OSR groups by the associated Kenward–Roger *F*-statistic. Specifically, for each PC regression model, the *F*-statistics of the saturated model were expressed as a percentage of the sum of all *F*-statistics for the PCs within each OSR group. To identify the minimal set of important PCs for determining seed yield, the above PC regression models were refined through a sequential backwards elimination process removing any term found to be non-significant (at a 5% threshold).

#### Partial Least Squares

Partial least square regression models were fitted to the subsets of WOSR and SOSR genotypes separately. Analyses were performed on all macrotraits (173 and 100 observations for WOSR and SOSR, respectively) and on alltraits (106 and 60 observations for WOSR and SOSR, respectively). Both the response (seed yield) and explanatory variables were standardised (mean centred and scaled by the standard deviation) and the PLS2 algorithm was used. Only observations with a complete set of measured traits were included.

#### Modelling Seed Number per Pod

A model was fitted to the SNPP to explore the relationship between SNPP and valve length. Generalised additive mixed models were fitted to the data using the gamm4 package in R (R Core Team, 2020; Wood and Scheipl, 2020). Random effects of glasshouse/(row × column) were included and a separate thin-plate regression spline was fitted to each OSR type.

#### Modelling Ovule Abortion

A simple linear regression with groups was fitted to ovule number vs. SNPP. The interaction term was dropped as it was non-significant. A generalised linear model was fitted to ovule abortion vs. SNPP with gamma distribution and identity link function. The linear predictor was a regression with groups including interaction term. A simple linear regression with groups, including interaction term, was fitted to ovule abortion vs. valve length. Terms were assessed via *F*-statistics (linear model) or Chi-squared tests of deviance (generalised linear model).

Linear mixed models (both univariate and PC regressions) and PLSs analysis was done using Genstat 20th Edition (VSN International Ltd., Hertfordshire, United Kingdom). PCs analysis, linear models, generalised linear models and generalised additive mixed models were done using R statistical software environment v3.6.1.

## Results

### Seed Yield

**FIGURE 1:**
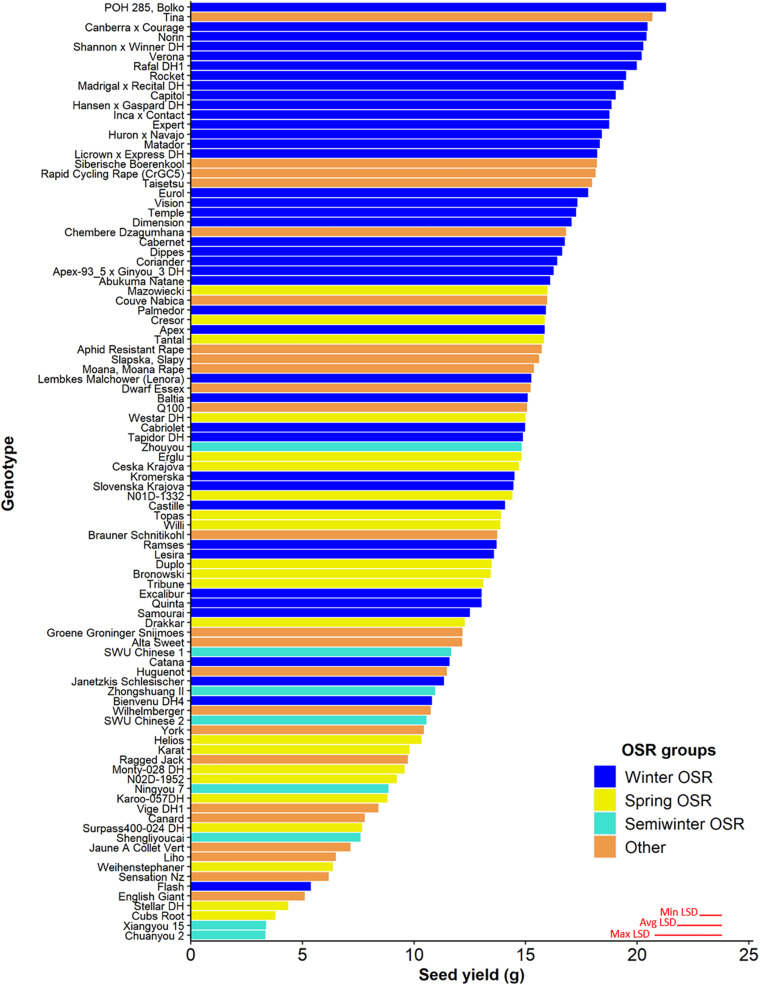
Seed yield (g) for the 96 genotypes of the *Brassica napus* diversity set population for the 4 OSR groups (Winter OSR, Spring OSR, Semiwinter OSR, and Other). Data are the mean of five biological replicates. Maximum, average, and minimum least significant difference (max LSD, avg LSD, and min LSD, respectively) are represented as red lines in the bottom right corner of the graph.

**FIGURE 2:**
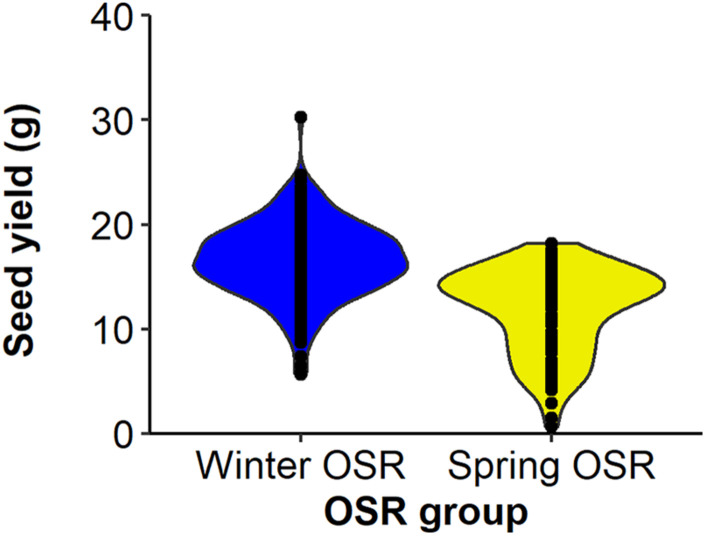
Distribution of seed yield (g) for Winter OSR and Spring OSR (*n* = 5). Points represent the individual observations for the genotypes in each group.

Seed yield was measured for the whole diversity set population (Figure 1), presenting values from 3.3–21.3 g per plant. The four OSR groups in which the population was divided (see section “Materials and Methods”) did not show an even distribution of seed yield (sequential *F*_3,329_ = 99.33, *P* < 0.001), with further differences in seed yield observed between lines within each group (*F*_92,275_ = 6.01, *P* < 0.001). WOSR and Other groups presented the highest seed yields within the population. The fact that some genotypes within the Other group presented high seed yield was quite surprising, as these lines are not selected for seed yield, but for their edible leaves or roots. POH 285, Bolko presented the highest yield not only for WOSR, but also for the whole population, meanwhile Tina had the highest seed yield from the Other group. Flash and English Giant were the genotypes with the lowest seed yield for WOSR and Other groups. Mazowiecki and Tapidor DH were the best yielders for SOSR and Semiwinter OSR group, respectively. Meanwhile, Chuanyou 2 and Xiangyou 15 were the genotypes which presented the lowest seed yield not only for Semiwinter OSR group, but for the whole population. Both WOSR and SOSR genotypes are bred for seed yield. Although both groups presented a similar range of seed yield, a high density of SOSR genotypes occur within a slightly lower narrow range than WOSR (Figure 2). A summary statistics over the whole population of plants including the mean, standard deviation (SD), the minimum (Min), maximum (Max), coefficient of variation (%CV), and heritability can be found at Supplementary Table 3. The predicted means of all the measured traits can be found at Supplementary Table 4.

### Seed Yield Components

To break down the seed yield trait and determine the interrelation between its components, rank correlations were calculated at macrotrait and alltraits level with a main focus in WOSR and SOSR groups. Pod length was separated into valve and beak length to estimate the contribution of these two phenotypic traits to seed yield. Similarly, gynoecia length was split as ovary and style length. For WOSR_macro_ we found positive correlations between seed yield and seed number (*r* = 0.87) and oil content (*r* = 0.61), with total seed number showing the strongest positive correlation with seed yield (Supplementary Figure 1A). For SOSR_macro_ we found positive correlations between seed yield and seed number (*r* = 0.89), oil content (*r* = 0.85), SNPP_*M*_ (*r* = 0.70), valve length (*r* = 0.59), pod length (*r* = 0.59), number of pods on a secondary inflorescence (*r* = 0.53) and number of pods in the main inflorescence (*r* = 0.48), and negative correlations between seed yield and TGW (*r* = −0.49), seed area (*r* = −0.5) and seed area coefficient of variation (*r* = −0.56) (Supplementary Figure 1B). SOSR presented higher correlations between seed yield and oil content and SNPP_*M*_ compared to WOSR. For alltraits we observed weaker correlations between seed yield and its components (Supplementary Figure 2). Nevertheless, different patterns were noticed between macrotraits and microtraits. SNPP presented a higher positive correlation with ovule number for SOSR_alltraits_ than for WOSR_alltraits_ (*r* = 0.36 vs. 0.16). A negative correlation between total seed number and ovule number was observed for WOSR_alltraits_ (*r* = −0.10), while a positive correlation was observed for SOSR_alltraits_ (*r* = 0.39). We also observed some positive and negative correlations at microtraits level, with ovule number presenting a negative and positive correlation with seed yield for WOSR_alltraits_ and SOSR_alltraits_ (*r* = −0.12 and 0.35) respectively. Hence, the differences in the interrelations between the seed yield components observed in both OSR groups, as well as against seed yield, suggested varying contributions of these phenotypic traits to seed yield. Correlation coefficients for Semiwinter OSR and Others group can be found at Supplementary Figures 3, 4.

### Comparison of Principal Component Contribution to Seed Yield Between WOSR and SOSR

The whole diversity set population was included in a PCA as it had a good representation of OSR cultivars that exploit historical recombination between molecular markers and loci associated with traits relevant to seed yield (Harper et al., 2012; Havlickova et al., 2018). This approach enabled us to have an unbiased study at a whole population level. Subsequently, a principal component regression analysis against seed yield was performed to compare the contribution of each PC to seed yield for each OSR group as a percentage of total variation explained from all PCs (expressed as contribution to yield (%) herein). Each PC identified combinations of the measured traits explaining the maximal variation in the data, defining ideal reproductive strategies that plants adopt within the population for macrotraits and alltraits, respectively (Supplementary Files 1, 2). We observed different contribution of PC to seed yield in all groups. As WOSR and SOSR are major seed yielders, we focused our efforts in analysing the differences between these groups. For macrotraits, 12 PCs were identified explaining 95.46% of the variation in the phenotypic traits with associated contribution to seed yield given in Table 2. PC1_macro_ was the reproductive strategy that presented the highest contribution to seed yield in WOSR and SOSR, being the most important reproductive strategy followed by both groups. However, PC1_macro_ contributed ∼1.5-fold more to seed yield in SOSR than in WOSR (78.67 vs. 54.63%). PC5_macro_ was the next most important reproductive strategy contributing to seed yield for both WOSR and SOSR, but in this case, it explained ∼1.6-fold more contribution to seed yield in WOSR than in SOSR. We observed that PC6_macro_, PC7_macro_, and PC10_macro_ were also contributing to seed yield, albeit more substantially in WOSR compared to SOSR, for which seed yield was largely explained by PC1_macro_ alone. For alltraits, 16 PCs were identified explaining 95.96% of the variation in the phenotypic data with associated contribution to seed yield given in Table 3. Similarly to macrotraits, PC1_alltraits_ was the most important reproductive strategy in both WOSR and SOSR, explaining ∼1.7-fold more contribution to seed yield in SOSR. PC7_alltraits_ and PC6_alltraits_ were the next most relevant reproductive strategies in WOSR and SOSR, presenting a similar contribution to seed yield within each OSR groups but again, explaining more contribution to seed yield in WOSR than in SOSR. For both macrotraits and alltraits, reproductive strategies contributed more to seed yield in WOSR compared to SOSR, for which seed yield was largely explained by PC1_macro_ and PC1_alltraits_.

**TABLE 2:** Principal component (PC) contribution to seed yield as a percentage of total variation explained from all PCs for Winter OSR and Spring OSR [expressed as contribution to seed yield (%)] for macrotraits.

Winter OSR		Spring OSR	
	
PCs_macro_	Contribution to seed yield (%)	PCs_macro_	Contribution to seed yield (%)
PC1_macro_	54.63	PC1_macro_	78.67
PC2_macro_	2.96	PC2_macro_	0.52
PC3_macro_	0.02	PC3_macro_	0.56
PC4_macro_	0.91	PC4_macro_	0.07
PC5_macro_	13.11	PC5_macro_	8.39
PC6_macro_	6.80	PC6_macro_	6.38
PC7_macro_	8.46	PC7_macro_	2.33
PC8_macro_	2.48	PC8_macro_	0.02
PC9_macro_	0.43	PC9_macro_	0.34
PC10_macro_	9.16	PC10_macro_	2.68
PC11_macro_	1.01	PC11_macro_	0.02
PC12_macro_	0.05	PC12_macro_	0.05

*Percentage is calculated as the ratio of the *F*-statistic of each PC divided by the sum of *F*-statistics for all PCs within each OSR group. PCs with small contributions to seed yield are observed. Each PC represents combinations of the measured traits, defining ideal reproductive strategies that plants adopt within the population. Supplementary File 1 contains the combination of traits for each PC.*

**TABLE 3:** Principal component (PC) contribution to seed yield as a percentage of total variation explained from all PCs for Winter OSR and Spring OSR [expressed as contribution to yield (%)] for alltraits (macro and microtraits together).

Winter OSR		Spring OSR	
	
PC_alltraits_	Contribution to seed yield (%)	PC_alltraits_	Contribution to seed yield (%)
PC1_alltraits_	46.11	PC1_alltraits_	76.45
PC2_alltraits_	1.30	PC2_alltraits_	0.81
PC3_alltraits_	0.68	PC3_alltraits_	0.40
PC4_alltraits_	1.39	PC4_alltraits_	1.05
PC5_alltraits_	0.29	PC5_alltraits_	1.13
PC6_alltraits_	10.06	PC6_alltraits_	6.08
PC7_alltraits_	12.75	PC7_alltraits_	6.59
PC8_alltraits_	1.77	PC8_alltraits_	0.02
PC9_alltraits_	6.05	PC9_alltraits_	2.76
PC10_alltraits_	8.31	PC10_alltraits_	1.16
PC11_*a1ltraits*_	1.02	PC11_*a1ltraits*_	0.03
PC12_alltraits_	0.44	PC12_alltraits_	0.00
PC13_alltraits_	4.31	PC13_alltraits_	0.72
PC14_alltraits_	3.80	PC14_alltraits_	2.19
PC15_alltraits_	1.69	PC15_alltraits_	0.09
PC16_alltraits_	0.03	PC16_alltraits_	0.51

*Percentage is calculated as the ratio of the *F*-statistic of each PC divided by the sum of *F*-statistics for all PCs within each OSR group. PCs with small contributions to seed yield are observed. Each PC represents combinations of the measured traits, defining ideal reproductive strategies that plants adopt within the population. Supplementary File 2 contains the combination of traits for each PC.*

### Identification of the Most Significant Reproductive Strategies Contributing to Seed Yield Within WOSR and SOSR

As described in Tables 2, 3, there was a total of 12 and 16 PCs for macrotraits and alltraits, respectively, that contribute to seed yield to a larger or smaller extent. To refine this further, a sequential elimination of non-significant terms in the PC regression enabled the identification and order of the most significant reproductive strategies contributing to seed yield within WOSR and SOSR group at macrotraits and alltraits level (Table 4). For macrotraits, WOSR presented nine PCs, meanwhile SOSR showed seven PCs that contributed significantly to seed yield.

**TABLE 4:** Reproductive strategies (PCs) that significantly contribute to seed yield in Winter OSR and Spring OSR for macrotraits and for alltraits (macro and microtraits together) when dropping terms.

Macrotraits				Alltraits			
	
Winter OSR		Spring OSR		Winter OSR		Spring OSR	
				
PC order	Approximate *F*-statistics	PC order	Approximate *F*-statistics	PC order	Approximate *F*-statistics	PC order	Approximate *F*-statistics
PC1_macro_	363.61	PC1_macro_	413.66	PC1_alltraits_	235.2	PC1_alltraits_	289.69
PC5_macro_	117.95	PC5_macro_	71.05	PC7_alltraits_	68.6	PC7_alltraits_	54.71
PC7_macro_	71.39	PC6_macro_	33.59	PC6_alltraits_	50.1	PC6_alltraits_	35.23
PC10_macro_	56.01	PC10_macro_	16.58	PC10_alltraits_	38.14	PC14_alltraits_	14.08
PC6_macro_	44.8	PC7_macro_	15.97	PC14_alltraits_	29.46	PC9_alltraits_	9.98
PC8_macro_	22.22	PC3_macro_	4.77	PC9_alltraits_	26.77	PC5_alltraits_	8.54
PC2_macro_	20.16	PC2_macro_	1.63	PC2_alltraits_	22.49	PC4_alltraits_	8.17
PC11_macro_	8.52			PC13_alltraits_	15.73	PC13_alltraits_	6.42
PC4_macro_	6.38			PC15_alltraits_	9.35	PC10_alltraits_	4.94
				PC8_alltraits_	8.72		
				PC4_alltraits_	7.81		

*The order of importance of the reproductive strategies for yield and the approximate *F*-statistics are reported in the table. Each PC represents combinations of the measured traits, defining ideal reproductive strategies that plants adopt within the population.*

As before, PC1_macro_ was the main reproductive strategy for both WOSR and SOSR, followed by PC5_macro_. For WOSR PC7_macro_ and PC10_macro_ were the next most relevant reproductive strategies contributing to seed yield, whereas PC6_macro_ and PC10_macro_ were the next reproductive strategies for SOSR. For alltraits, WOSR presented 11 reproductive strategies meanwhile we observed 9 for SOSR. While PC1_alltraits_, PC7_alltraits_, and PC6_alltraits_ were the first three reproductive strategies for both OSR groups, WOSR presented PC10_alltraits_ while SOSR presented PC14_alltraits_ as important reproductive strategies contributing to seed yield. The higher number of significant PCs by WOSR at both macrotraits and alltraits level confirmed that WOSR presented more reproductive strategies to explain seed yield compared to SOSR. The difference in the number of reproductive strategies observed in WOSR could be due to the fact that this OSR group had a larger set of genotypes than SOSR. However, we observed that the same reproductive strategies present a different order of importance for seed yield between OSR groups implying different reproductive strategies are being followed.

### Reproductive Strategies Observed in the Population for Macrotraits and Alltraits

Here, we present the most important and significant reproductive strategies contributing to seed yield that plants adopt within the diversity set population. We highlighted the combination or trade-offs for the two and three most important reproductive strategies contributing to seed yield of the measured macrotraits and alltraits, respectively (Tables 5, 6). Moreover, the other significant PCs contributing to seed yield with a small contribution to seed yield not covered in this section for WOSR and SOSR for macrotraits and alltraits can be found at Supplementary Files 1, 2, respectively. At the macrotrait level, the main reproductive strategy followed by WOSR and SOSR was PC1_macro_, it being the most important strategy followed by both OSR groups. This reproductive strategy was associated with a reduced number of secondary inflorescences, whereby plants focused their energy and resources mainly in the main inflorescence, and in few secondary branches (Table 5). This strategy was also associated with a high number of pods in the main inflorescence and in secondary inflorescences, presenting a low percentage of pod abortion at the whole plant level. These plants produced long pods in the main inflorescence with a large number of seeds within them. The plants produced a large number of small seeds and with high oil content. Overall, this strategy was associated with high seed yield, with seed number at the whole plant level being the most important trait contributing to seed yield. The next most relevant reproductive strategy (PC5_macro_) was associated with plants producing more flowers in the whole plant, long pods with large uniform circular seeds, but with more seed area coefficient of variation. As in the main reproductive strategy (PC1_macro_), this strategy was associated with high seed oil content. However, in this case, seed area was more important than seed number.

**TABLE 5:** Winter OSR and Spring OSR reproductive strategies for macrotraits.

Reproductive strategy	Positively correlated with seed yield	Negatively correlated with seed yield
PC1_macro_	SN (6.70%) SNPP_*M*_ (6.48%) OC (5.50%) VL_*M*_ (5.07%) PL_*M*_ (5.01%) SW_*M*_ (4.93%) PN_*M*_ (4.45%) PN_1S_ (3.91%)	PA (6.17%) PA_*S*_ (5.92%) PA_1s_ (5.90%) PA_*M*_ (5.88%) NI (4.77%) NI-1 (4.66%) FN (4.05%) TGW (3.90%)
PC5_macro_	SW_*M*_ (6.39%) SC_*M*_ (6.07%) TGW (5.62%) OC (5.51%) FN (5.39%) SC (4.73%) SA_*M*_ (4.67%) PL_*M*_ (4.52%) VL_*M*_ (4.42%) SA (4.09%)	SAvar (7.55%)

*The traits in the tables are ordered from the most to the least influential trait within the reproductive strategy. The contribution to the PC for each trait is also included, calculated as the percentage of each loading, only showing those traits with an important contribution.*

**TABLE 6:** Winter OSR and Spring OSR reproductive strategies for alltraits (macro and microtraits together).

Reproductive strategy	Positively correlated with seed yield	Negatively correlated with seed yield
PC1_alltraits_	SNPP_*M*_ (5.92%) SN (5.82%) VL_*M*_ (4.84%) PL_*M*_ (4.82%) OC (4.66%) SW_*M*_ (4.63%) PN_*M*_ (4.11%) PN_1S_ (3.53%) BL (3.46%) PN (2.37%) ON (1.80%)	PA (5.70%) PA_*M*_ (5.64%) PAs (5.54%) PA1s (5.46%) NI (4.27%) NI-1 (4.14%) FN (3.88%) TGW (3.20%)
PC7_alltraits_	ON (7.90%) SN (6.84%) OL (6.83%) GL (6.67%) OC (5.90%) PA (3.97%) PA_*S*_ (3.89%) FN (3.76%)	BL (4.91%) SC (4.18%)
PC6_alltraits_	TGW (6.08%) SW_*M*_ (5.38%) SA_*M*_ (5.33%) SA (4.82%) FN (4.50%) PL_*M*_ (4.39%) VL_*M*_ (4.16%) BL (3.88%) OC (3.85%)	SAvar (5.16%) OL (4.00%) TF (3.45%) OAvar (3.26%) GL (3.22%) ON (2.35%)

*The traits in the tables are ordered from the most to the least influential trait within the reproductive strategy. The contribution to PC for each trait is also included, calculated as the percentage of each loading. In this case, traits with lower contributions were also included in order to elucidate relationships with microtraits.*

The analysis was extended to include microtraits to assess whether these traits significantly influenced the macrotraits and or seed yield (Table 6). The main reproductive strategy for both WOSR and SOSR including alltraits (PC1_alltraits_) was similar to PC1_macro_, but in this case it was also associated with plants presenting long beaks and a high number of ovules in the main inflorescence. The next reproductive strategy (PC7_alltraits_) was associated with plants with short beaks but with high number of ovules, long ovaries and long gynoecia, with these traits presenting a high contribution within the reproductive strategy. These plants produced a high number of flowers and displayed pod abortion. This strategy was associated with plants generating a large number of seeds with high seed oil content. Finally, the next most relevant reproductive strategy (PC6_alltraits_) was similar to PC5_macro,_ with the addition of being associated with short ovaries and gynoecia, low number of ovules, long beaks, and seeds with high oil content.

### High Yielders Follow Several Reproductive Strategies

Winter OSR and SOSR genotypes were ranked for each reproductive strategy for macrotraits and alltraits in order to identify whether consistently high yielding OSR follow a certain strategy. WOSR genotypes POH 285, Bolko; Canberra × Courage, Norin, Shannon × Winner DH, and Verona and SOSR genotypes Mazowiecki, Cresor, Tantal, Westar DH, and Erglu were identified as high yielders. We observed that high yielders in both OSR groups did not follow a particular reproductive strategy for macrotraits or alltraits, but a combination of them, as suggested by our results (Supplementary Tables 5–8). However, consistent with our analyses, they all presented a good rank for PC1_macro_ and PC1_alltraits_.

Interestingly, the five worst WOSR yielders Flash, Bienvenu DH4, Catana, Samourai, and Quinta showed low adoption of the main reproductive strategy PC1 in both macrotraits and alltraits level. For SOSR, as observed in WOSR, the worst five worst yielders, Cubs Root, Stellar DH, Weihenstephaner, Surpass400-024DH, and Karoo-057-DH also presented a low rank for PC1_macro_ and PC1_alltraits_.

### A PLS Analysis Corroborates the Main Strategy for WOSR and SOSR, and Seed Number Is the Best Predictor of Seed Yield

Our initial analyses at the whole population level highlighted a distinctive response between WOSR and SOSR in terms of reproductive strategies relevant to seed yield. Subsequently, WOSR and SOSR groups were analysed separately to fully capture the strategies employed by each. A PLS analysis for WOSR and SOSR was performed in order to corroborate the results obtained at whole population level and to determine the best predictor of seed yield for WOSR and SOSR, respectively. The PLS approach iteratively identifies combinations of traits, defining the PLS components that are maximally related to seed yield and then combines these components to get an overall assessment of the contribution of each trait to seed yield. For the macrotraits, nine components explained 96.3 and 97.3% of the variation in seed yield in WOSR and SOSR, respectively (Supplementary Table 9). We observed that although both OSR groups presented the same number of components (chosen by cross-validation), the contribution to seed yield from component 1 was substantially higher in SOSR, explaining 74% of the variation in seed yield. Component 1 presented the same combination of significant traits for PC1_macro_ and PC1_alltraits_, confirming that this was the main reproductive strategy contributing to high seed yield at both macrotraits and alltraits level. Component 1 also presented the highest variation in seed yield for WOSR (44.2%), but other components were also represented to a large extent, observing more reproductive strategies for optimising seed yield in this set of 42 WOSR genotypes than in the 22 SOSR genotypes. However, the different order of importance presented by these two OSR groups suggests that different reproductive traits are more influential for one group than in the other. The same trends and results were observed for alltraits. For WOSR, 11 components explained 97.0% of the variation on seed yield, while 8 components explained 96.8% of the variation in seed yield for SOSR (Supplementary Table 10).

Taking account of all the components contributing to seed yield, the most important trait affecting seed yield in WOSR and SOSR for macrotraits and WOSR alltraits was seed number, followed by TGW, both positively associated with yield. On the other hand, the predictors most negatively associated with seed yield were number of flowers, number of pods on secondary inflorescences and number of secondary inflorescences in WOSR. Whereas for SOSR they were time to flowering, pod abortion in the whole plant and seed compactness from 10 pods from the main inflorescence (Supplementary Tables 11, 12).

### The Number of Seeds per Pod Increases as Valve Lengthens

**FIGURE 3:**
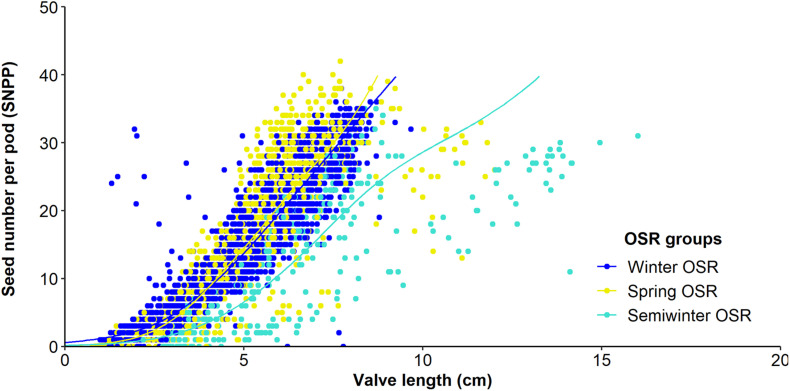
Relationship between seed number/pod (SNPP) and valve length from 10 pods from the main inflorescence for Winter OSR, Spring OSR, and Semiwinter OSR. Fitted lines are the result of a generalised additive mixed model.

**FIGURE 4:**
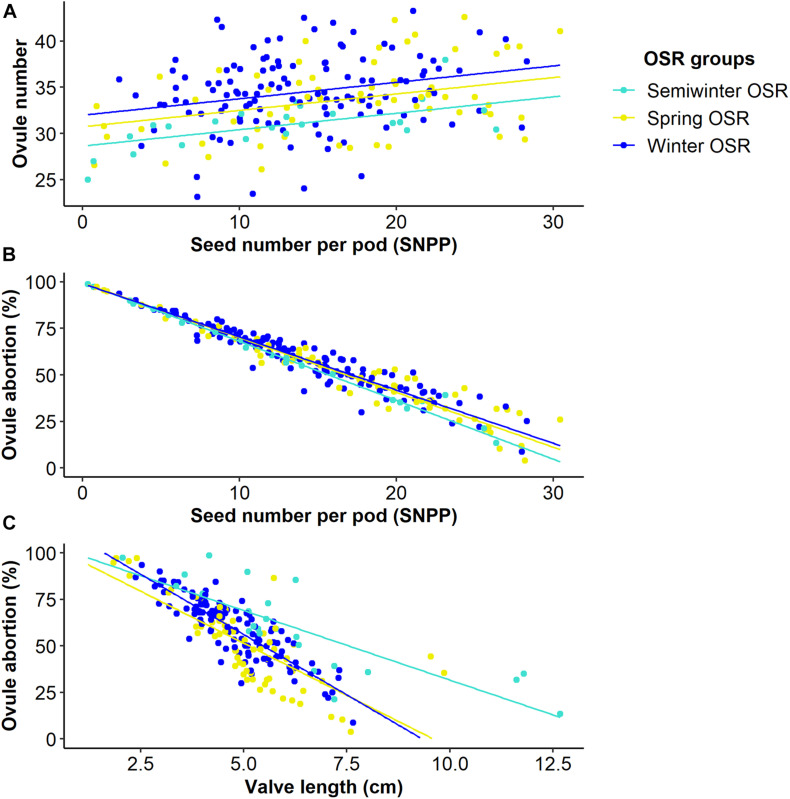
Relationships between seed number per pod (SNPP) and valve length and two microtraits in Winter OSR, Spring OSR, and Semiwinter OSR (*n* = 3). **(A)** Linear regression for ovule number vs. SNPP, **(B)** generalised linear model for ovule abortion vs. SNPP, **(C)** linear regression for ovule abortion vs. valve length.

As seed number was the best predictor of seed yield, and SNPP and pod length presented a high contribution in the main reproductive strategy followed by WOSR and SOSR, we investigated whether the number of seeds increased as the pod valves lengthen (Figure 3). We observed that as valve length increased, the SNPP increased following a similar pattern in WOSR and SOSR, presenting an exponential increase until approximately 5 cm of valve length, followed by a more linear increase. We observed the same trend for all SOSR genotypes with one exception, Karat. Interestingly, Semiwinter OSR genotypes presented, in general, long valves with fewer seeds, which was especially evident in Xiangyou 15 and Zhongshuang II. This highlights the fact that the selection of long pods needs to be linked to good seed packing. On the other hand, we observed some WOSR genotypes, such as Kromerska and Hansen × Gaspard DH, that presented shorter valves with a high SNPP. Interestingly, Hansen × Gaspard DH also presented long valves with a low number of seed, and in particular this genotype exhibited a high variability in SNPP. There is a clear relationship between SNPP and the number of ovules across all three OSR groups, with SNPP increasing as the number of ovules increases (*P* < 0.001, *F*_1,276_ = 29.7) (Figure 4A). Further there was a difference in the overall number of ovules (Significant intercept, *P* < 0.001, *F*_3,276_ = 7.4) with WOSR having the highest number of ovules followed by SOSR and Semiwinter OSR. On the other hand, ovule abortion is inversely related to SNPP (Significant trend of *X*_1_ = 4765.4, *P* < 0.001), presenting an indistinguishable trend for WOSR and SOSR, but presenting a steeper trend in Semiwinter OSR with a smaller % of ovule abortion observed for the same SNPP for Semiwinter OSR (significant interaction of *X*_3_ = 8.8, *P* = 0.03) (Figure 4B). Finally, we also observed an inverse relationship between ovule abortion and valve length (significant trend *F*_1,273_ = 553.3, *P* < 0.001) across all three OSR groups, although the strength of this relationship differed across the groups (significant interaction *F*_3,273_ = 10.1, *P* < 0.001) (Figure 4C). In particular, the steepest relationship was observed in WOSR, although in general SOSR showed a lower % of ovule abortion. Meanwhile, Semiwinter OSR had long valves regardless of the percentage of ovule abortion. Interestingly, Karat seems to be following the same pattern as the Semiwinter OSR, trend that we already observed in Figure 3.

**FIGURE 5:**
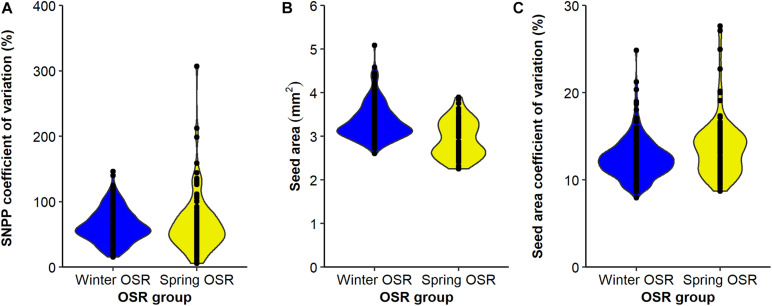
Distribution of **(A)** seed number/pod (SNPP) coefficient for variation (%), **(B)** seed area (mm^2^), and **(C)** seed area coefficient of variation (%) for Winter OSR and Spring OSR (*n* = 5). Points represent the individual observations for the genotypes in each group.

The SNPP coefficient of variation presented a wider distribution for SOSR compared to WOSR (Figure 5A), but on average, both groups presented no significant differences for this trait (sequential *F*_1.339_ = 0.92, *P* = 0.337), demonstrating that this trait is as variable in both OSR groups. In general, WOSR genotypes presented bigger seed areas than SOSR (sequential *F*_1.318_ = 151.84, *P* < 0.001, Figure 5B), presenting a maximum around 3.2 mm^2^ with a skewness towards bigger seeds. However, SOSR genotypes seemed to produce two types of seeds, one around 2.7 mm^2^ and other around 3.5 mm^2^, presenting a multimodal distribution. Finally, SOSR produced less uniform seed areas compared to WOSR (sequential *F*_1.313_ = 21.02, *P* < 0.001, Figure 5C).

## Discussion

The differences in seed yield observed for the OSR groups in the diversity set population can be explained by varying combinations of reproductive strategies adopted by these groups, including the importance of the microtraits to seed yield. Our analyses highlighted distinct differences in the contribution to seed yield arising from different reproductive strategies, with PC1_macro_ and PC1_alltraits_ providing the biggest contribution to seed yield, especially evident in SOSR. In this strategy, the seeds from the main inflorescence were the principal source of seed yield for WOSR and SOSR. This strategy was also associated with a reduced number of secondary inflorescences, presumably with the plants relocating their carbon assimilates primarily to the main inflorescence, producing a certain amount of long pods with a large number of small seeds within them. This result highlights the importance that plant architecture may play in assimilate partitioning among plant organs. The successful development of pods and seeds and their variation in number is determined by the quantity of assimilates available at the whole plant level and the competition with other developing organs (Arathi et al., 1999; Diepenbrock, 2000). This is particularly crucial during the plant reproductive phase, when competition between developing pods and seeds among different inflorescences occurs, causing a high demand of carbon assimilates within a short period of time (Wang et al., 2011). Consequently, the reduction of number of flowering inflorescences decreases intra-plant competition that may be responsible for loss of buds, flowers and seeds (Diepenbrock, 2000), resulting in a high number of pods in the main inflorescence with reduced percentage of pod abortion and enhanced seed yield. Leaves are the major source of photosynthesis in OSR until flowering, providing assimilate source supporting pod growth. At the onset of flowering, leaf area decreases due to canopy shading and flower photon reflectivity and leaves start to fall, reducing leaf photosynthesis by 40% (Diepenbrock, 2000). Therefore, long pods enhance photosynthetic capacity as the developing pod walls become the main intercept of solar radiation, contributing up to 70% of the assimilates to seed filling (Diepenbrock, 2000; Li et al., 2019). This is in concordance with our results, in which we observed that longer valves can support the development of a higher number of seeds. Previous studies have also found that number of pods per plant and SNPP in *Brassica* sp. genotypes were major contributors to seed yield (Özer et al., 1999; Badaran et al., 2007; Tunçtürk and Çiçti, 2007; Lu et al., 2011; Chen et al., 2014; Ul-Hasan et al., 2014; Hu et al., 2017a; Moradi et al., 2017; Ahmadzadeh et al., 2019; Tariq et al., 2020). Specifically, Başalma (2008) also reported that the number of pods in the main inflorescence rather than the whole plant presented a positive correlation with seed yield in WOSR. However, our study not only confirms that seed number is the single most important trait affecting seed yield, but also identifies the architecture of the plant and the interplay of developmental traits followed by SOSR and WOSR as the main reproductive strategy to high seed yield. Within this main reproductive strategy that both OSR groups were following, we observed a trade-off between seed number and seed size, as the plants had a high number of seeds at the expense of seed size, as observed in other studies (Kirkegaard et al., 2018). This can again be explained by resource availability in the mother plant, with plasticity in seed number proving more beneficial in an environment of variable resource availability (Sadras, 2007). Interestingly, when the microtraits were included in the analyses, we observed that long beaks and a high number of ovules were also associated with the main reproductive strategy described above, highlighting the importance of these often-ignored phenotypic traits. Not only a high number of ovules is essential to obtain a final high number of seeds, the trait affecting seed yield maximally in the main reproductive strategy, but also its fertilisation and correct development. It is of crucial importance to understand the factors affecting ovule and seed abortion to understand how these traits are affecting seed number and valve length, and hence seed yield. We observed that ovule abortion is inversely correlated with valve length, but the mechanisms underlying this relationship remain unknown. It may be that a reduction of available assimilates during the differentiation of the ovary could lead to fewer ovules capable of fertilisation in shorter pods. The fact that ovule abortion decreases when longer valves develop is in concordance with the development of more SNPP in longer pods as mentioned above (Diepenbrock, 2000; Li et al., 2019). Longer pods present a greater photosynthetic green area, accumulating more photo-assimilates which can favour the development of the fertilised ovules into seeds while the pods are expanding by influencing the expression of genes involved in reserve synthesis and metabolism, which affect seed filling, size, and weight (Li et al., 2019). Different hormonal levels between genotypes with long and short pods may also explain the observed trend between ovule abortion and valve length. Auxins, gibberellins, cytokinins, and salicylic acid are phytohormones with a known role in seed development and pod growth (De Bouille et al., 1989; Bennett et al., 2011; Cao et al., 2020; Tang et al., 2020), and a change in the ratio of these may account for the differences observed in ovule abortion between pods of varying sizes.

Although the reproductive strategy explained above (PC1) was the main reproductive strategy for both OSR groups, other reproductive strategies presented significant contribution to seed yield albeit to a lesser extent. These strategies highlighted the importance of the main inflorescence by producing long pods with big seeds at the macrotraits level. When the microtraits were included, we observed the importance of producing a high number of ovules with long ovaries and gynoecia at expense of beak length and seed compactness for one strategy (PC7_alltraits_) or generating long pods with big seeds with short ovary and gynoecia lengths (PC6_alltraits_). These strategies highlight the importance of the microtraits along with the macrotraits, and how these affect the plant architecture and the final seed yield. For example, smaller ovaries can lead to smaller seeds or less amount of bigger seeds due to a reduced ovary space. Hence, understanding these developmental processes and how they translate to seed yield will provide novel insight for increasing seed yield in crop species. Although the above reproductive strategies presented less contribution to seed yield than PC1, the fact that WOSR retained more of these strategies compared to SOSR in both macrotraits and alltraits level was an important difference between these two OSR groups, which can be associated to their different life cycles. WOSR requires vernalisation to promote the onset of flowering, being grown largely in Western Europe and United Kingdom, where winters are mild. Their seeds are sown in later summer and survive winter in a leaf rosette form, putting a lot of effort in vegetative growth. They flower between March and May, completing the development of pod and seeds by the end of June (Diepenbrock, 2000; Nesi et al., 2008; Brown et al., 2019). On the other hand, SOSR genotypes present a faster life cycle and are cultivated in Canada, Asia, and Eastern Europe. In these countries, winters are too cold and SOSR genotypes are sown at the end of winter as they are not vernalisation dependent (Snowdon et al., 2007; Nesi et al., 2008). The differences in the life cycle and temperatures the plants are subject to may explain the varying reproductive strategies, as WOSR cultivars experience more variable environmental conditions during their life cycle. Moreover, as its life cycle is longer than the SOSR, they have more time to remobilise the reserves from pre-flowering period (Riffkin et al., 2016) and to adapt and compensate for environmental or mechanical damages; as for example frost events at the onset of flowering (Lardon and Triboi-Blondel, 1995), periods of high temperatures during flowering that can cause a reduction in pollen viability and germinability and pod abortion (Angadi et al., 2000; Young et al., 2004) or water stress during flowering (Champolivier and Merrien, 1996; Elferjani and Soolanayakanahally, 2018); and hence secure reproductive success. The plasticity presented by WOSR may explain why WOSR genotypes have higher seed yields than SOSR.

The PLS analysis corroborated that the main inflorescence was the main contributor to seed yield in both OSR groups (PC1), and that although PC1 was the single larger reproductive strategy in WOSR contributing to seed yield, and among the studied genotypes, WOSR presented more reproductive strategies in order to explain seed yield. The most important traits contributing to seed yield in PLS components 2 and 3 after having accounted for the association between the components and seed yield, highlighted common traits with the significant reproductive strategies contributing to seed yield. Furthermore, seed number was the best predictor of seed yield for WOSR and SOSR, followed by TGW as a proxy of seed size, confirming the results observed at the whole population level.

**FIGURE 6:**
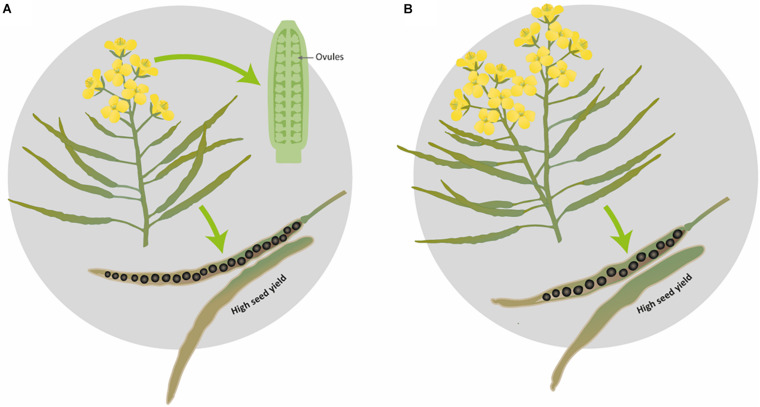
Graphical representation of the proposed ideotypes of *Brassica napus* for obtaining maximal seed yield. **(A)** Ideal ideotype for SOSR and WOSR, **(B)** additional WOSR ideotype leading to high seed yield.

In the present study, we propose two ideotypes for SOSR and WOSR for high seed yield. While we fully expect these association to hold, as this experiment was performed in controlled conditions with plants growing in pots, it would be interesting to confirm that these reproductive strategies would be followed in field conditions. Here, we propose that an ideal SOSR or WOSR phenotype (ideotype) should have a limited number of inflorescences with a good number of ovules and pods on the main inflorescence and reduced percentage of pod abortion. The pods should have long valves with high SNPP for producing seeds with high seed oil content (Figure 6A). The WOSR ideotype can also invest in more flowers, a few secondary inflorescences and bigger seeds in pods with long valves to produce high seed yields (Figure 6B). Both SNPP and pod length are phenotypic traits that present relatively high heritability, therefore are important targets for breeding selection (Shi et al., 2009; Zhang et al., 2011; Li et al., 2019) as they still present great variation in OSR germplasms resources. However, it is important to highlight that long pods in itself are not sufficient but should demonstrate good seed packing for maximal seed yield. It remains to be determined whether SNPP is subject to genetic control independent of ovule number. Nevertheless, understanding the biological process leading to a high number of ovules and hence, to a high number of seeds with a good seed packing will enable to harness knowledge to increase seed yield.

Our study uncovered that in spite of the genetic diversity represented across *Brassica* sp. genotypes, OSR follow primarily one discrete strategy for maximal seed yield, in which the main inflorescence is the principal source of seed yield, presenting a reduced number of secondary inflorescences and generating long valves with a high number of seeds in environmental controlled conditions of around 12 plants per m^2^ of plant population density. Although OSR plants demonstrate large differences in vernalisation, branching, flowering time, and canopy structure, they appear to uniformly prefer a single approach for seed yield. This knowledge is important for breeders in determining target traits for improvement that can confer maximum yield benefit in OSR.

## Data Availability Statement

The original contributions presented in the study are included in the article/Supplementary Material, further inquiries can be directed to the corresponding author.

## Author Contributions

SK conceived and supervised the project. KH designed the experiment and performed the statistical analyses. LS and CSG performed the experiments. LS analysed the data and wrote the manuscript with input from SK, PJE, and KH. All authors read and approved the final manuscript.

## Conflict of Interest

The authors declare that the research was conducted in the absence of any commercial or financial relationships that could be construed as a potential conflict of interest.

## Publisher’s Note

All claims expressed in this article are solely those of the authors and do not necessarily represent those of their affiliated organizations, or those of the publisher, the editors and the reviewers. Any product that may be evaluated in this article, or claim that may be made by its manufacturer, is not guaranteed or endorsed by the publisher.
