# Supplementary material for: Uncovering Trait Associations Resulting in Maximal Seed Yield in Winter and Spring Oilseed Rape
Source: Front Plant Sci. 2021 Sep 6;12:697576. doi: 10.3389/fpls.2021.697576 (PMC8450599; doi:10.3389/fpls.2021.697576)
Supplement: Supplementary file 1 [file Data_Sheet_1.PDF]

Supplemental Figure S1: Spearman's correlation for macrotraits for A) Winter OSR and B) Spring OSR groups (n=5). PH=plant height (cm), NI= number of flowering inflorescences, NI-1= number of secondary inflorescences, TF= time to flowering (days), FN= number of flowers on the whole plant, PN_M=number of pods on the main inflorescence, PN_{1S}=number of pods on a secondary inflorescence, PN_S=number of pods on secondary inflorescences, PN= number of pods on the whole plant, PA_M= pod abortion on the main inflorescence (%), PA_{1S}=pod abortion on a secondary inflorescence (%), PA_S=pod abortion in secondary inflorescences (%), PA=pod abortion in the whole plant (%), TM= time to maturity (days), PL_M=pod length from 10 pods from the main inflorescence (cm), VL_M= valve length from 10 pods from the main inflorescence (cm), $SNPP_M$= seed number/ pod from 10 pods from the main inflorescence, SA_M= seed area from 10 pods from the main inflorescence (mm^2), SC_M= seed compactness from 10 pods from the main inflorescence, SW_M= seed weight from 10 pods from the main inflorescence (g), SA= seed area from the whole plant (mm^2), SC= seed compactness from the whole plant, SAcvar= seed area coefficient of variation from whole plant (%), TGW= thousand grain weight (g), SN= estimated total seed number from the whole plant (by TGW), OC= seed oil content from the whole plant (%), SY= seed weight from the whole plant (seed yield, g).

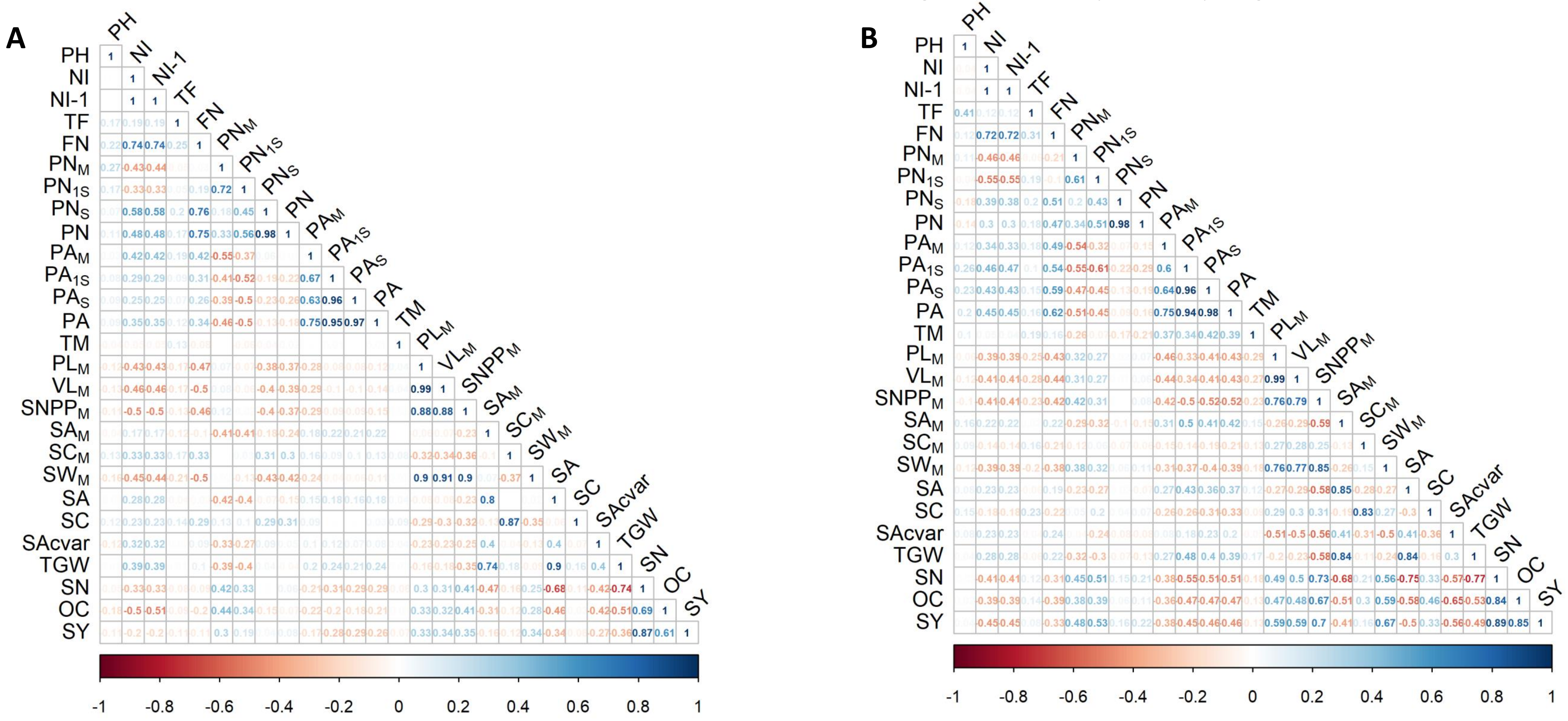

Supplemental Figure S2: Spearman's correlation for alltraits for A) Winter OSR and B) Spring OSR groups (n=3). PH=plant height (cm), NI= number of flowering inflorescences, NI-1= number of secondary inflorescences, TF= time to flowering (days), FN= number of flowers on the whole plant, ON=ovule number, OA=ovule area (mm^2), OAcvar=ovule area coefficient of variation (%), OL= ovary length (mm), GL=gynoecia length (mm), SL= style length (mm), PN_M=number of pods on the main inflorescence, PN_{1S}=number of pods on a secondary inflorescence, PN_S=number of pods on secondary inflorescences, PN= number of pods on the whole plant, PA_M= pod abortion on the main inflorescence (%), PA_{1S}=pod abortion on a secondary inflorescence (%), PA_S=pod abortion in secondary inflorescences (%), PA=pod abortion in the whole plant (%), TM= time to maturity (days), PL_M=pod length from 10 pods from the main inflorescence (cm), VL_M= valve length from 10 pods from the main inflorescence (cm), BL= beak length (cm), $SNPP_M$= seed number/ pod from 10 pods from the main inflorescence, SA_M= seed area from 10 pods from the main inflorescence (mm^2), SC_M= seed compactness from 10 pods from the main inflorescence, SW_M= seed weight from 10 pods from the main inflorescence (g), SA= seed area from the whole plant (mm^2), SC= seed compactness from the whole plant, SAcvar= seed area coefficient of variation from whole plant (%), TGW= thousand grain weight (g), SN= estimated total seed number from the whole plant (by TGW), OC= seed oil content from the whole plant (%), SY= seed weight from the whole plant (seed yield, g).

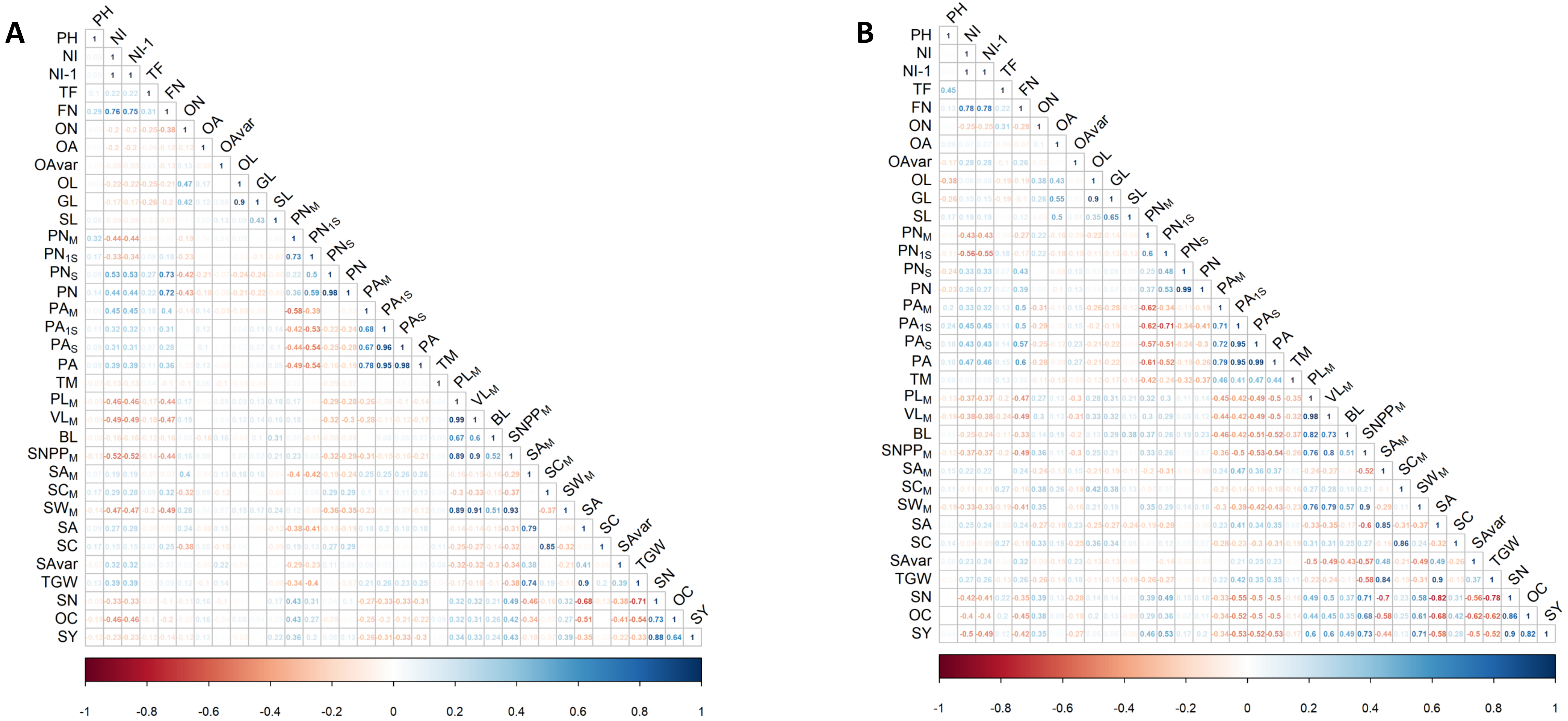

Supplemental Figure S3: Spearman's correlation for macrotraits for A) Semiwinter OSR and B) Others groups (n=5). PH=plant height (cm), NI= number of flowering inflorescences, NI-1= number of secondary inflorescences, TF= time to flowering (days), FN= number of flowers on the whole plant, PN_M=number of pods on the main inflorescence, PN_{1S}=number of pods on a secondary inflorescence, PN_S=number of pods on secondary inflorescences, PN= number of pods on the whole plant, PA_M= pod abortion on the main inflorescence (%), PA_{1S}=pod abortion on a secondary inflorescence (%), PA_S=pod abortion in secondary inflorescences (%), PA=pod abortion in the whole plant (%), TM= time to maturity (days), PL_M=pod length from 10 pods from the main inflorescence (cm), VL_M= valve length from 10 pods from the main inflorescence (cm), $SNPP_M$= seed number/ pod from 10 pods from the main inflorescence, SA_M= seed area from 10 pods from the main inflorescence (mm^2), SC_M= seed compactness from 10 pods from the main inflorescence, SW_M= seed weight from 10 pods from the main inflorescence (g), SA= seed area from the whole plant (mm^2), SC= seed compactness from the whole plant, SAcvar= seed area coefficient of variation from whole plant (%), TGW= thousand grain weight (g), SN= estimated total seed number from the whole plant (by TGW), OC= seed oil content from the whole plant (%), SY= seed weight from the whole plant (seed yield, g).

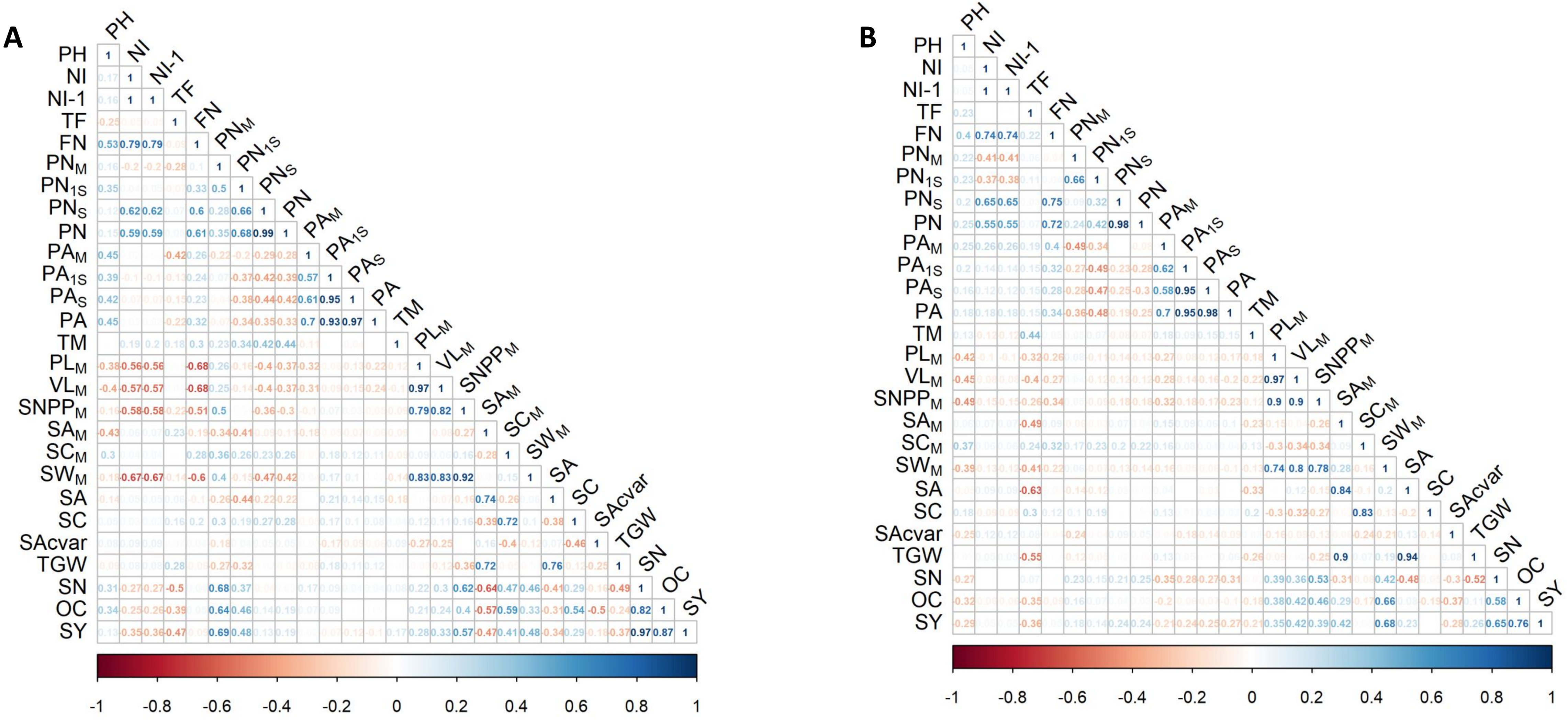

Supplemental Figure S4: Spearman's correlation for alltraits for A) Semiwinter OSR and B) Others groups (n=3). PH=plant height (cm), NI= number of flowering inflorescences, NI-1= number of secondary inflorescences, TF= time to flowering (days), FN= number of flowers on the whole plant, ON=ovule number, OA=ovule area (mm^2), OAcvar=ovule area coefficient of variation (%), OL= ovary length (mm), GL=gynoecia length (mm), SL= style length (mm), PN_M=number of pods on the main inflorescence, PN_{1S}=number of pods on a secondary inflorescence, PN_S=number of pods on secondary inflorescences, PN= number of pods on the whole plant, PA_M= pod abortion on the main inflorescence (%), PA_{1S}=pod abortion on a secondary inflorescence (%), PA_S=pod abortion in secondary inflorescences (%), PA=pod abortion in the whole plant (%), TM= time to maturity (days), PL_M=pod length from 10 pods from the main inflorescence (cm), VL_M= valve length from 10 pods from the main inflorescence (cm), BL= beak length (cm), $SNPP_M$= seed number/ pod from 10 pods from the main inflorescence, SA_M= seed area from 10 pods from the main inflorescence (mm^2), SC_M= seed compactness from 10 pods from the main inflorescence, SW_M= seed weight from 10 pods from the main inflorescence (g), SA= seed area from the whole plant (mm^2), SC= seed compactness from the whole plant, SAcvar= seed area coefficient of variation from whole plant (%), TGW= thousand grain weight (g), SN= estimated total seed number from the whole plant (by TGW), OC= seed oil content from the whole plant (%), SY= seed weight from the whole plant (seed yield, g).

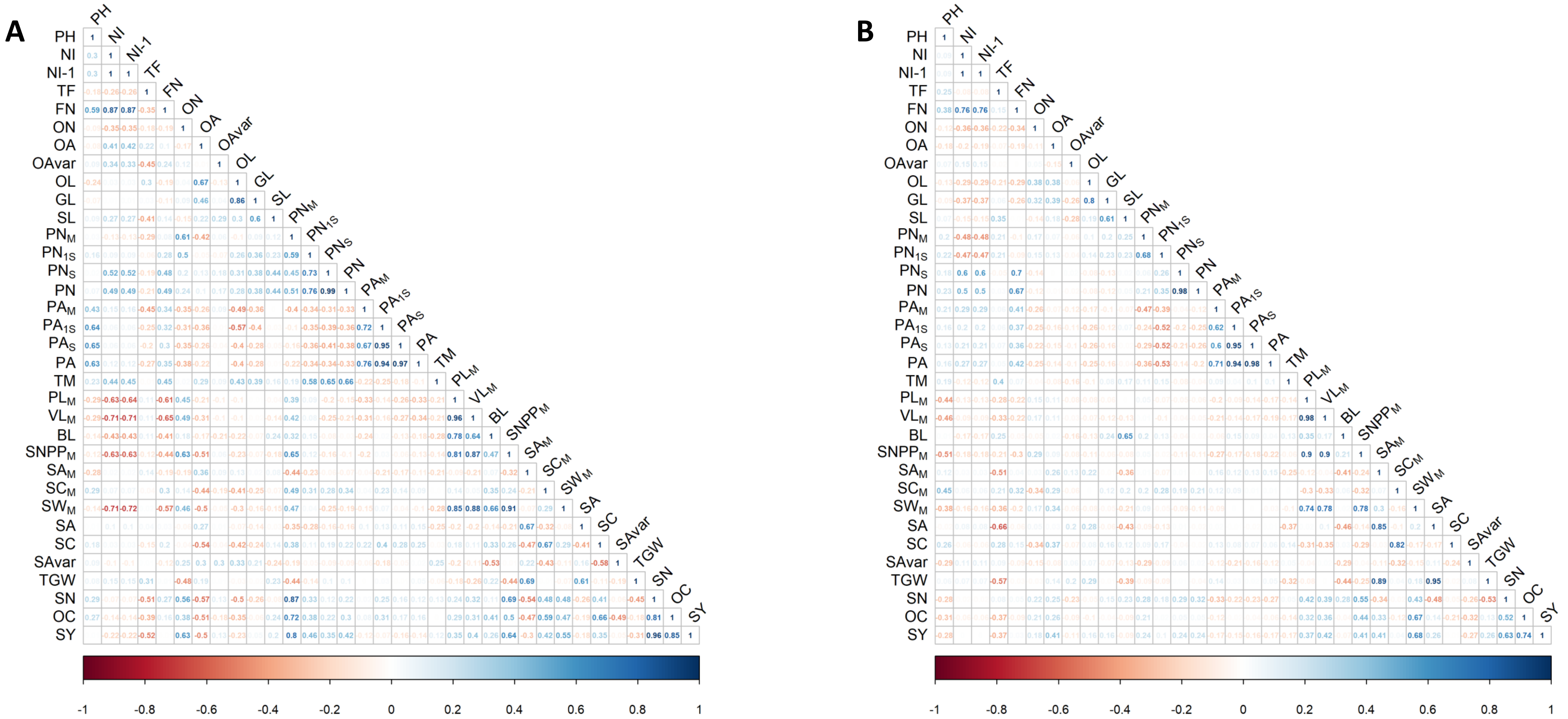

Supplementary File S1: Principal Component (PC) loadings for the significant macrotraits reproductive strategies retained by Winter OSR and Spring OSR groups.

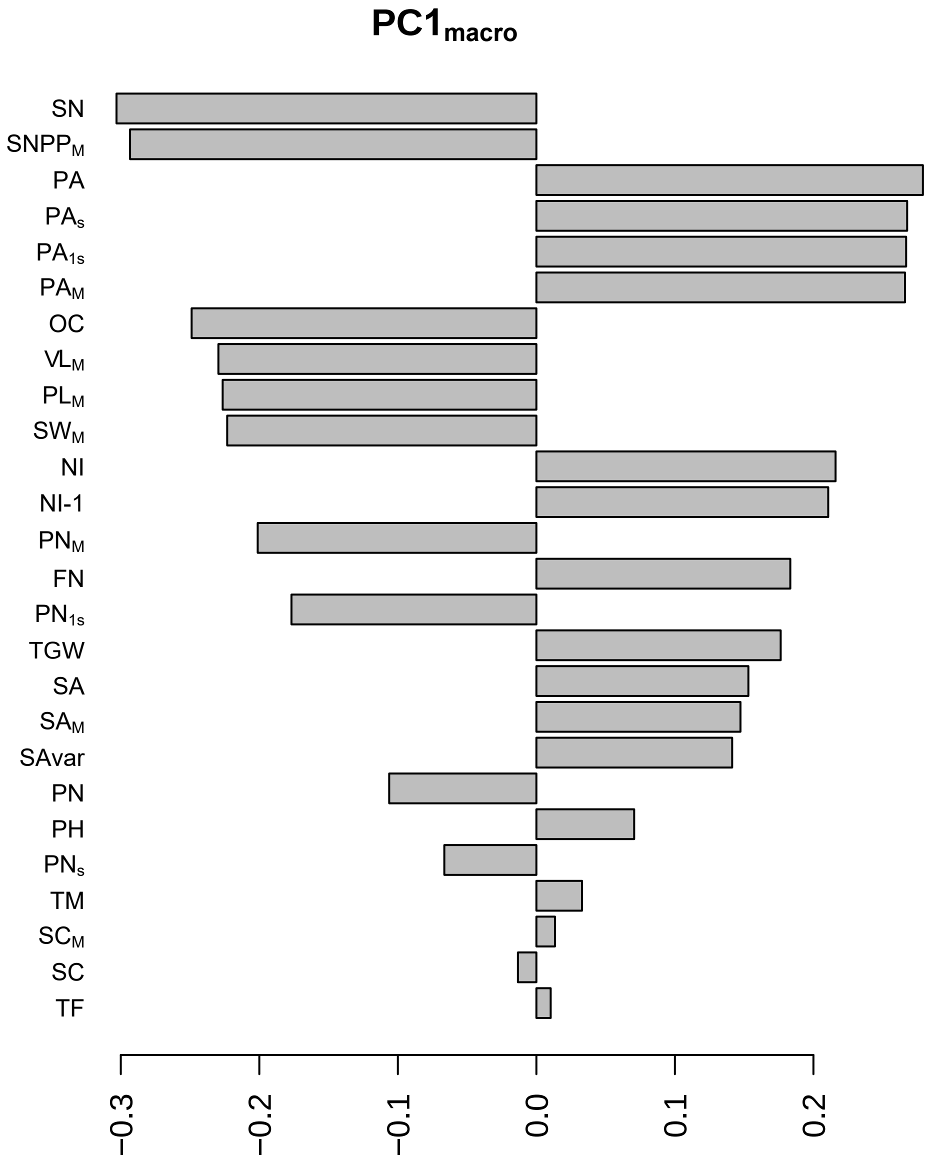

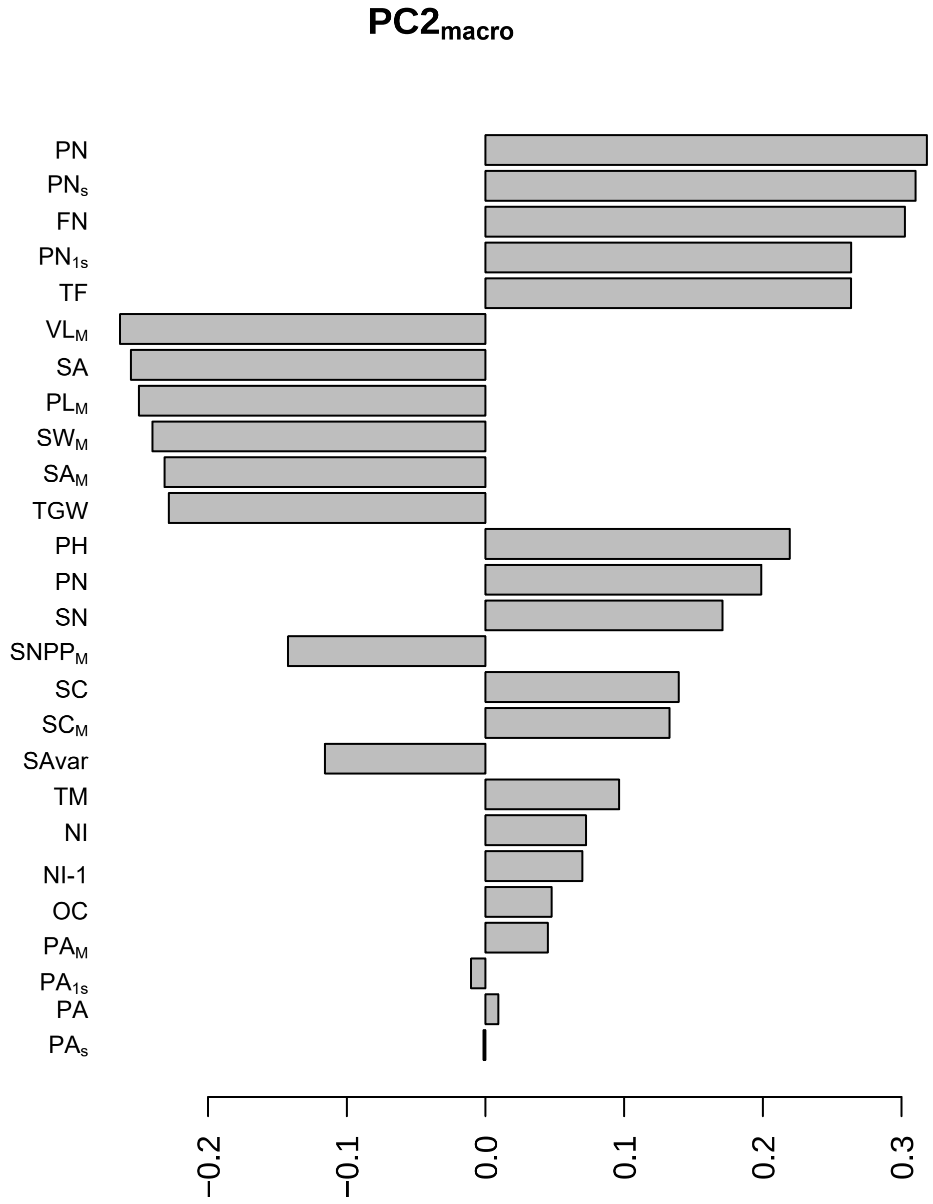

PC2macro
PN
PNs
FN
PN1s
TF
VLM
SA
PLM
SWM
SAM
TGW
PH
PN
SN
SNPPM
SC
SCM
SAvar
TM
NI
NI-1
OC
PAM
PA1s
PA
PAs
−0.2
−0.1
0.0
0.1
0.2
0.3

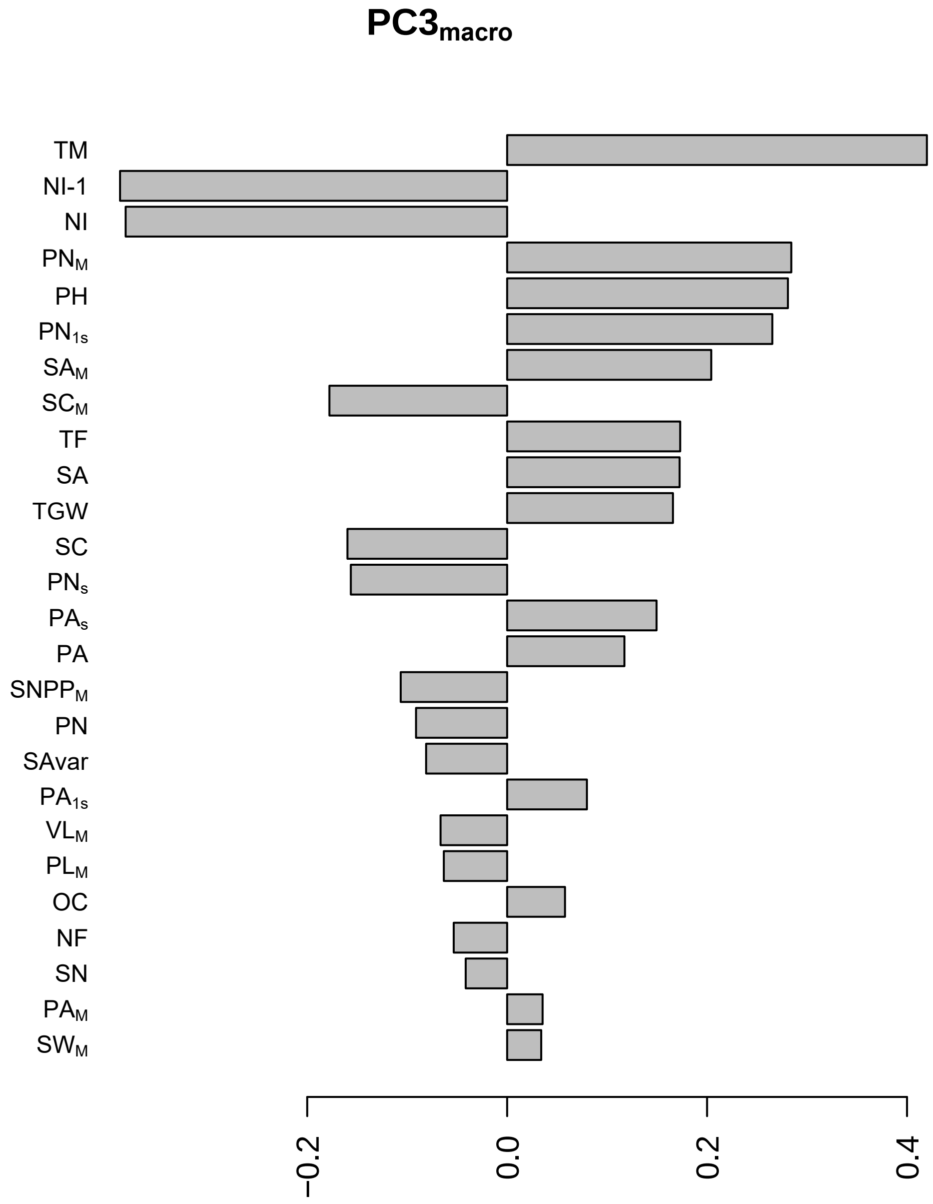
PC3macro
TM
NI-1
NI
PNM
PH
PN1s
SAM
SCM
TF
SA
TGW
SC
PNs
PAs
PA
SNPPM
PN
SAvar
PA1s
VLM
PLM
OC
NF
SN
PAM
SWM
−0.2
0.0
0.2
0.4

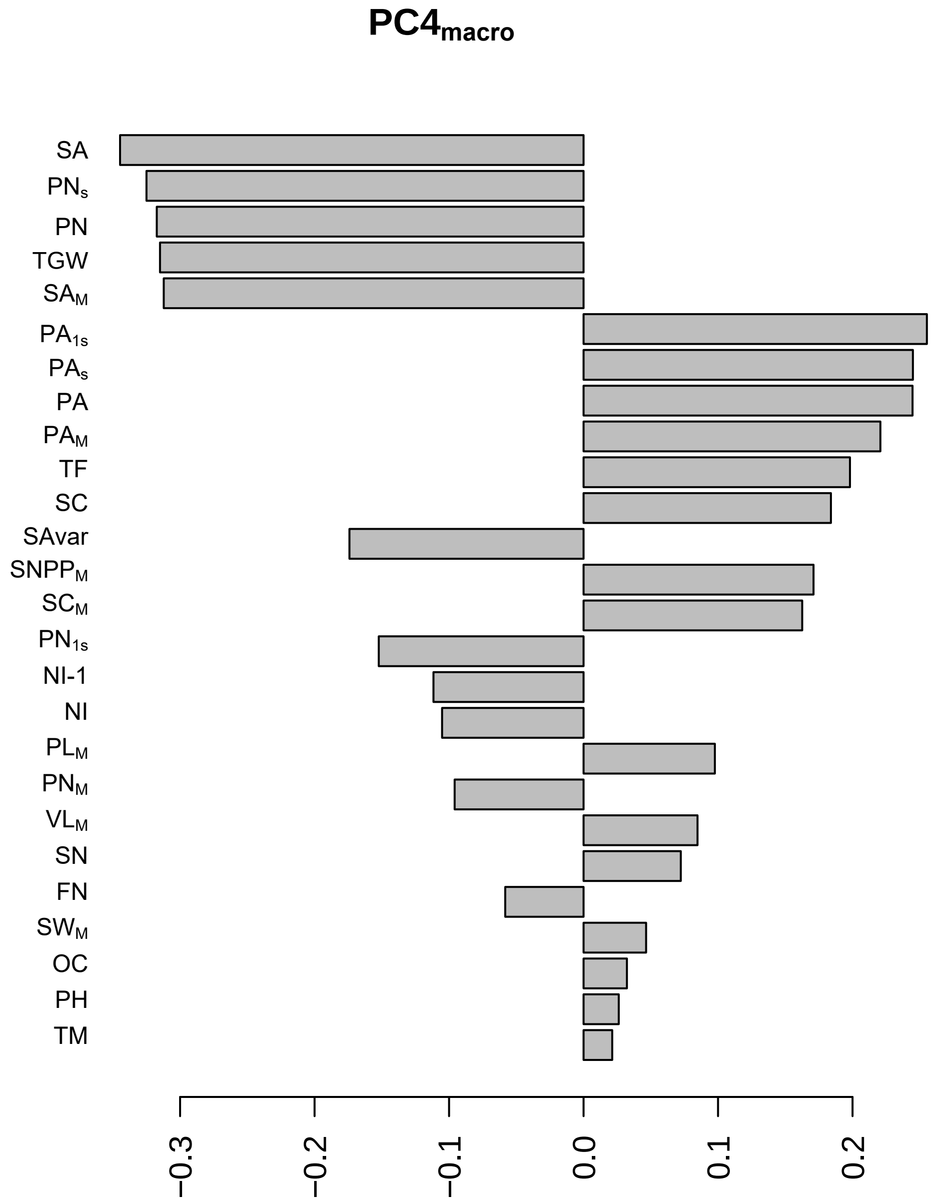
PC4macro
SA
PNs
PN
TGW
SAM
PA1s
PAs
PA
PAM
TF
SC
SAvar
SNPPM
SCM
PN1s
NI-1
NI
PLM
PNM
VLM
SN
FN
SWM
OC
PH
TM
−0.3
−0.2
−0.1
0.0
0.1
0.2

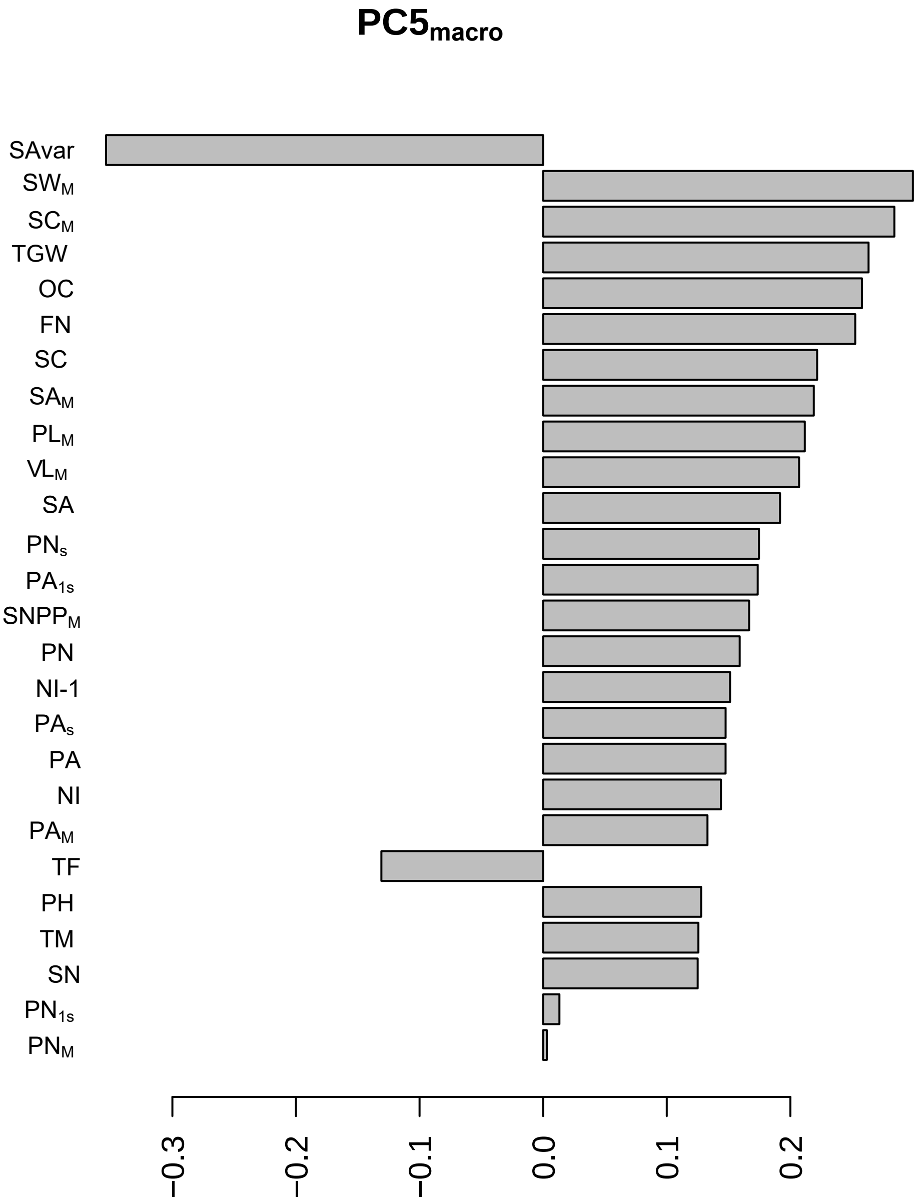

PC5macro
SAvar
SWM
SCM
TGW
OC
FN
SC
SAM
PLM
VLM
SA
PNs
PA1s
SNPPM
PN
NI-1
PAs
PA
NI
PAM
TF
PH
TM
SN
PN1s
PNM
−0.3
−0.2
−0.1
0.0
0.1
0.2

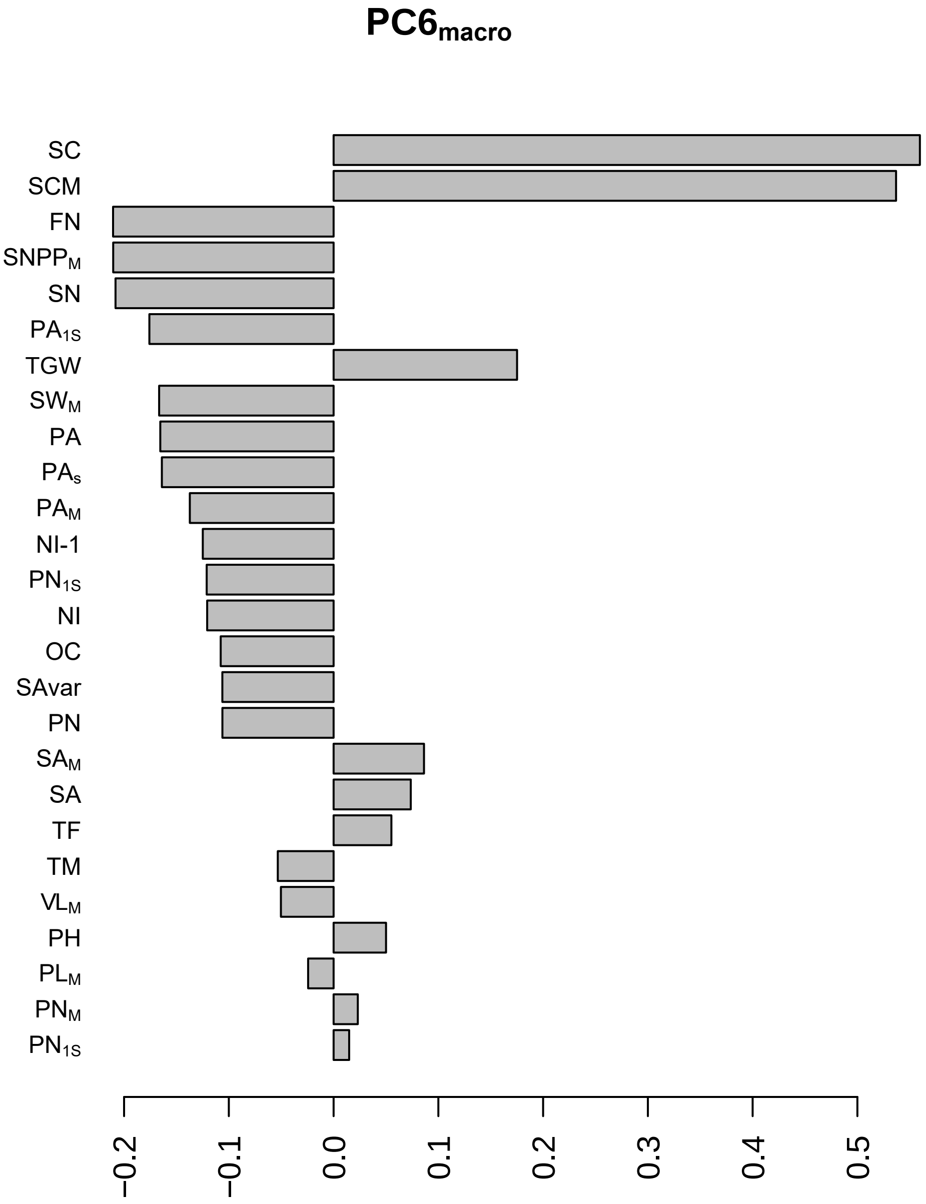

PC6macro
SC
SCM
FN
SNPPM
SN
PA1S
TGW
SWM
PA
PAs
PAM
NI-1
PN1S
NI
OC
SAvar
PN
SAM
SA
TF
TM
VLM
PH
PLM
PNM
PN1S
−0.2
−0.1
0.0
0.1
0.2
0.3
0.4
0.5

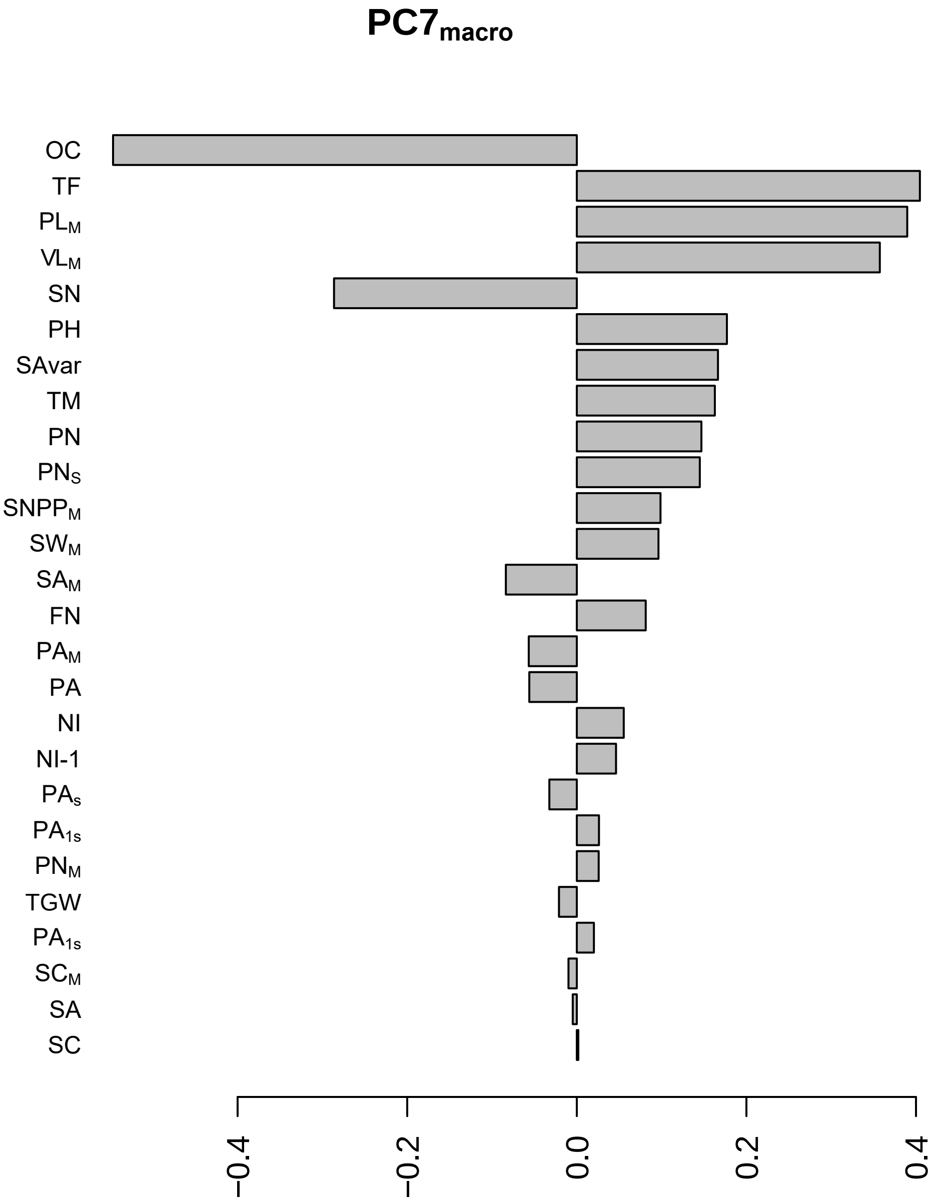
PC7 macro
OC
TF
PL M
VL M
SN
PH
SAvar
TM
PN
PN S
SNPP M
SW M
SA M
FN
PA M
PA
NI
NI-1
PA s
PA 1s
PN M
TGW
PA 1s
SC M
SA
SC
−0.4
−0.2
0.0
0.2
0.4

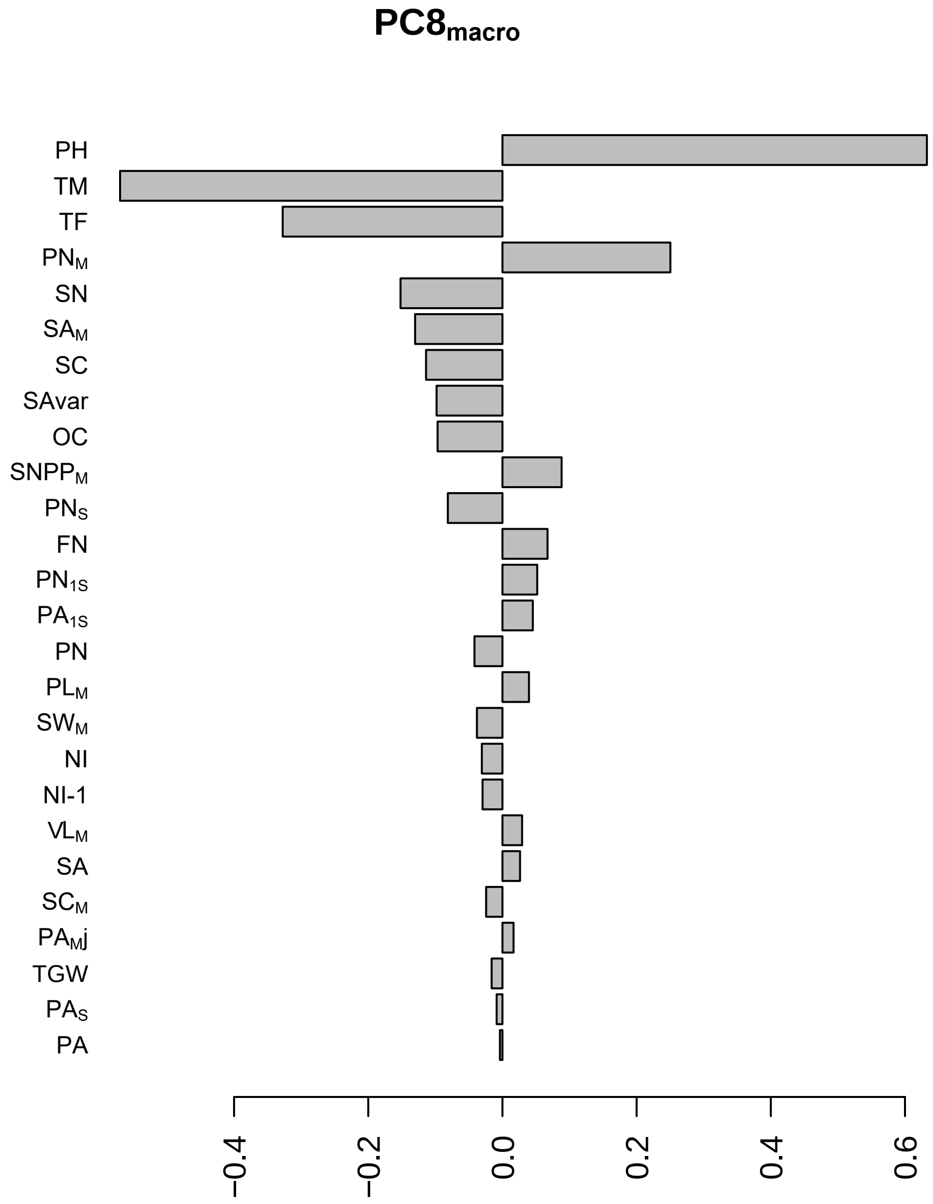

PC8macro
PH
TM
TF
PNM
SN
SAM
SC
SAvar
OC
SNPPM
PNS
FN
PN1S
PA1S
PN
PLM
SWM
NI
NI-1
VLM
SA
SCM
PAMj
TGW
PAS
PA
−0.4
−0.2
0.0
0.2
0.4
0.6

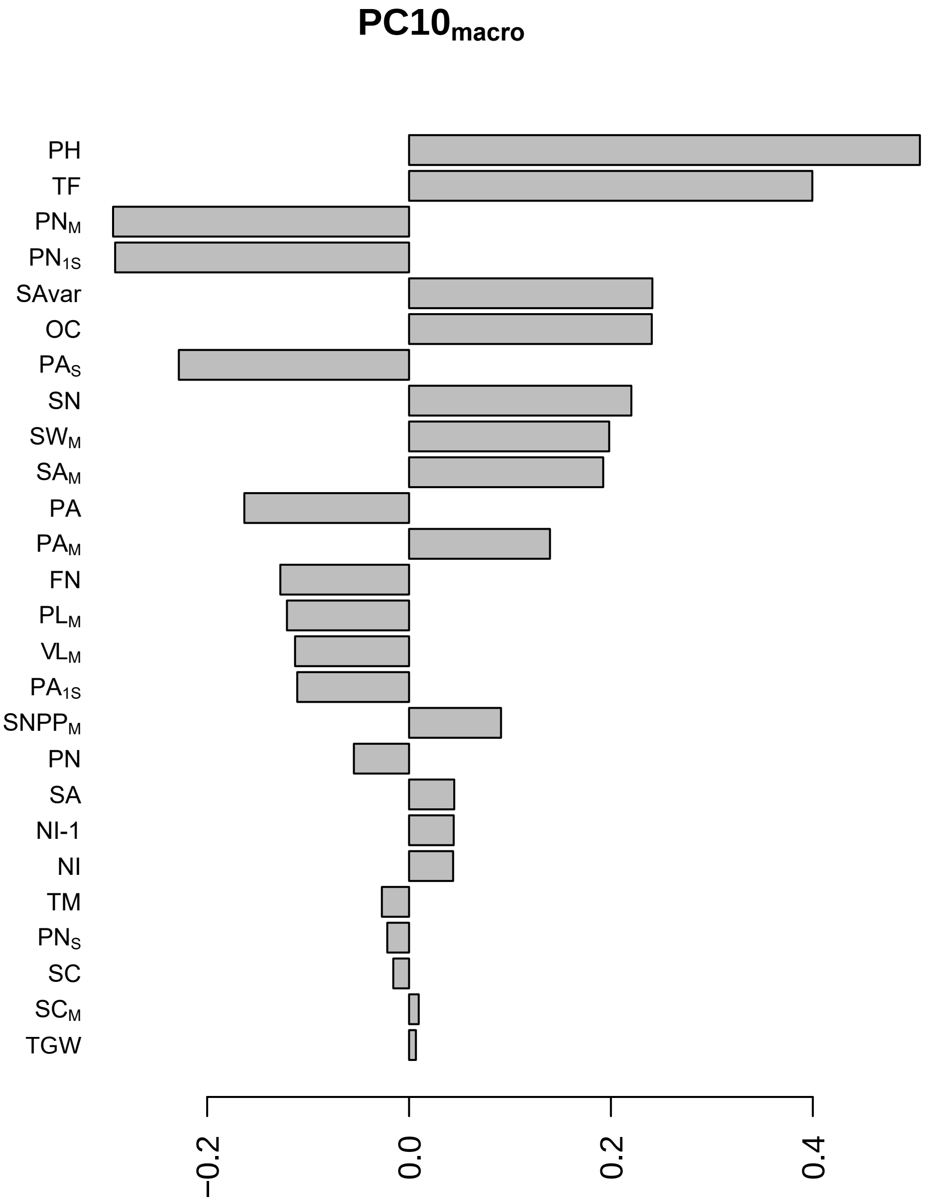

PC10macro
PH
TF
PNM
PN1S
SAvar
OC
PAS
SN
SWM
SAM
PA
PAM
FN
PLM
VLM
PA1S
SNPPM
PN
SA
NI-1
NI
TM
PNS
SC
SCM
TGW
−0.2
0.0
0.2
0.4

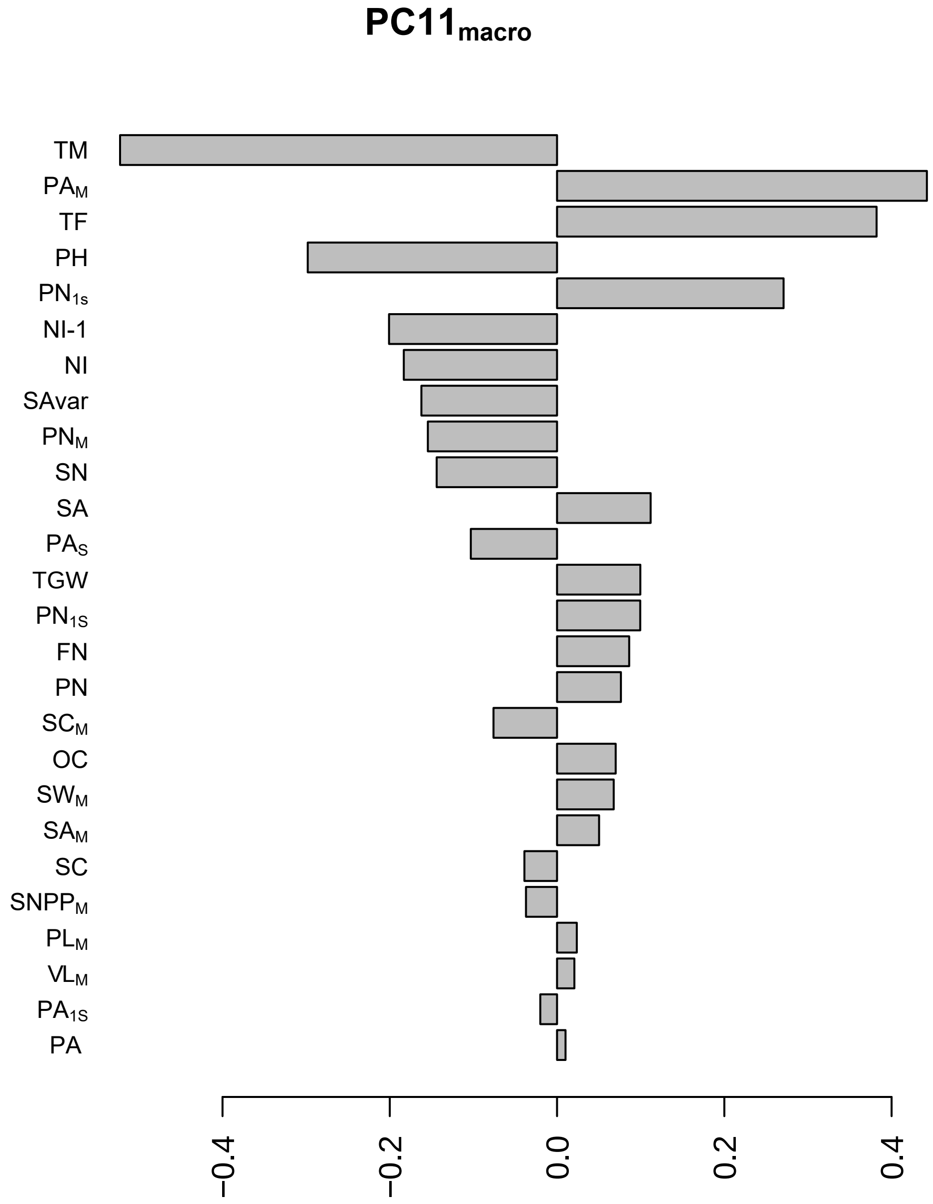

PC11macro
TM
PAM
TF
PH
PN1s
NI-1
NI
SAvar
PNM
SN
SA
PAS
TGW
PN1S
FN
PN
SCM
OC
SWM
SAM
SC
SNPPM
PLM
VLM
PA1S
PA
−0.4
−0.2
0.0
0.2
0.4

Supplementary File S2: Principal Component (PC) loadings for the significant alltraits reproductive strategies retained by Winter OSR and Spring OSR groups.

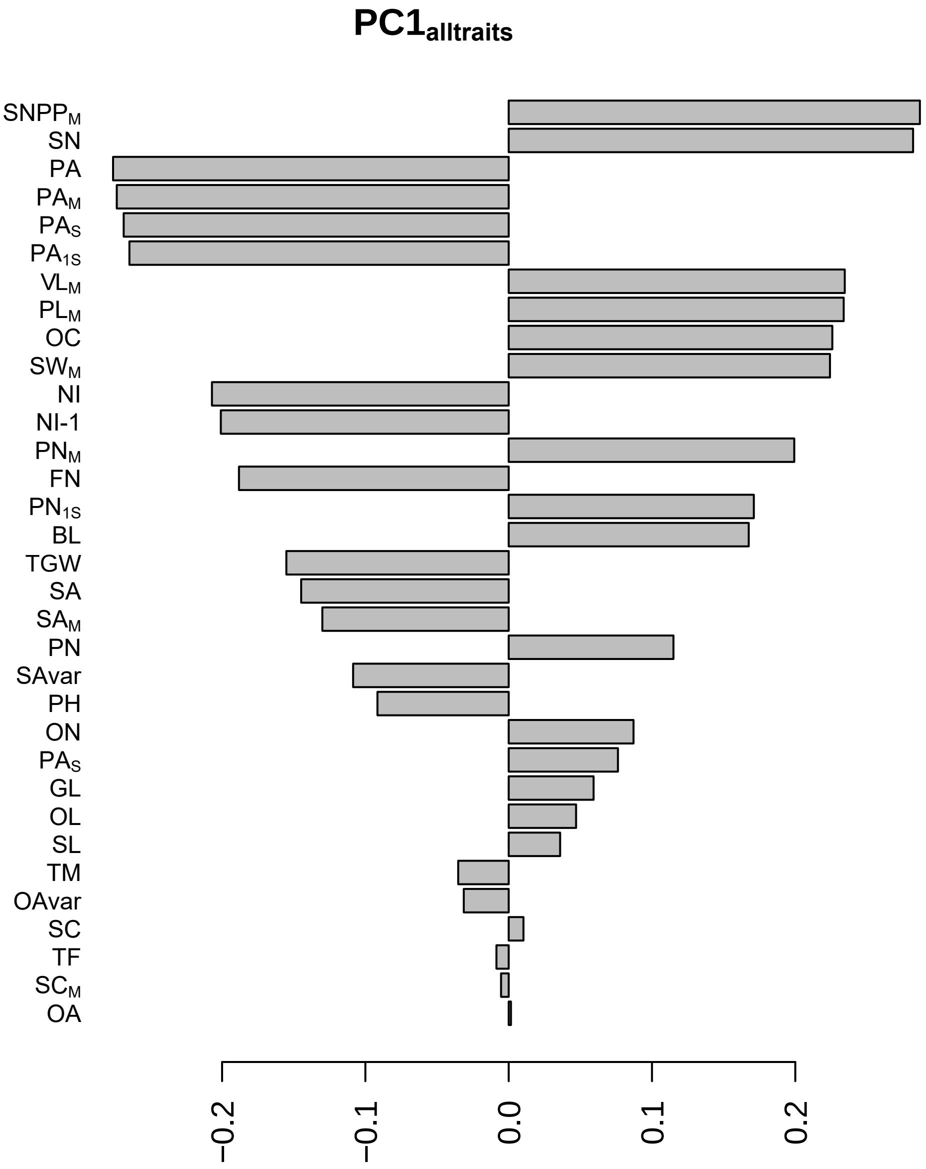

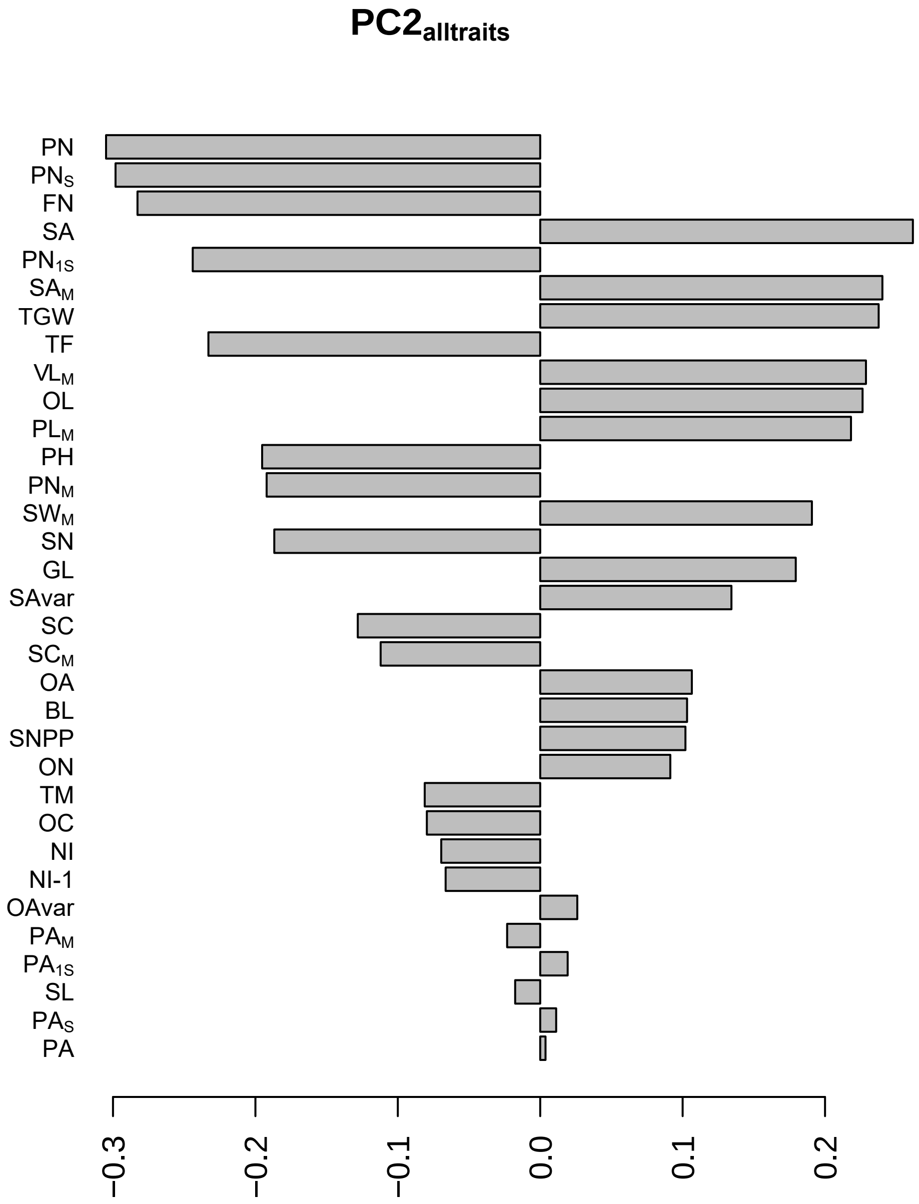

$PC2_{alltraits}$
PN
PN_S
FN
SA
PN_{1S}
SA_M
TGW
TF
VL_M
OL
PL_M
PH
PN_M
SW_M
SN
GL
SAvar
SC
SC_M
OA
BL
SNPP
ON
TM
OC
NI
NI-1
OAvar
PA_M
PA_{1S}
SL
PA_S
PA
−0.3
−0.2
−0.1
0.0
0.1
0.2

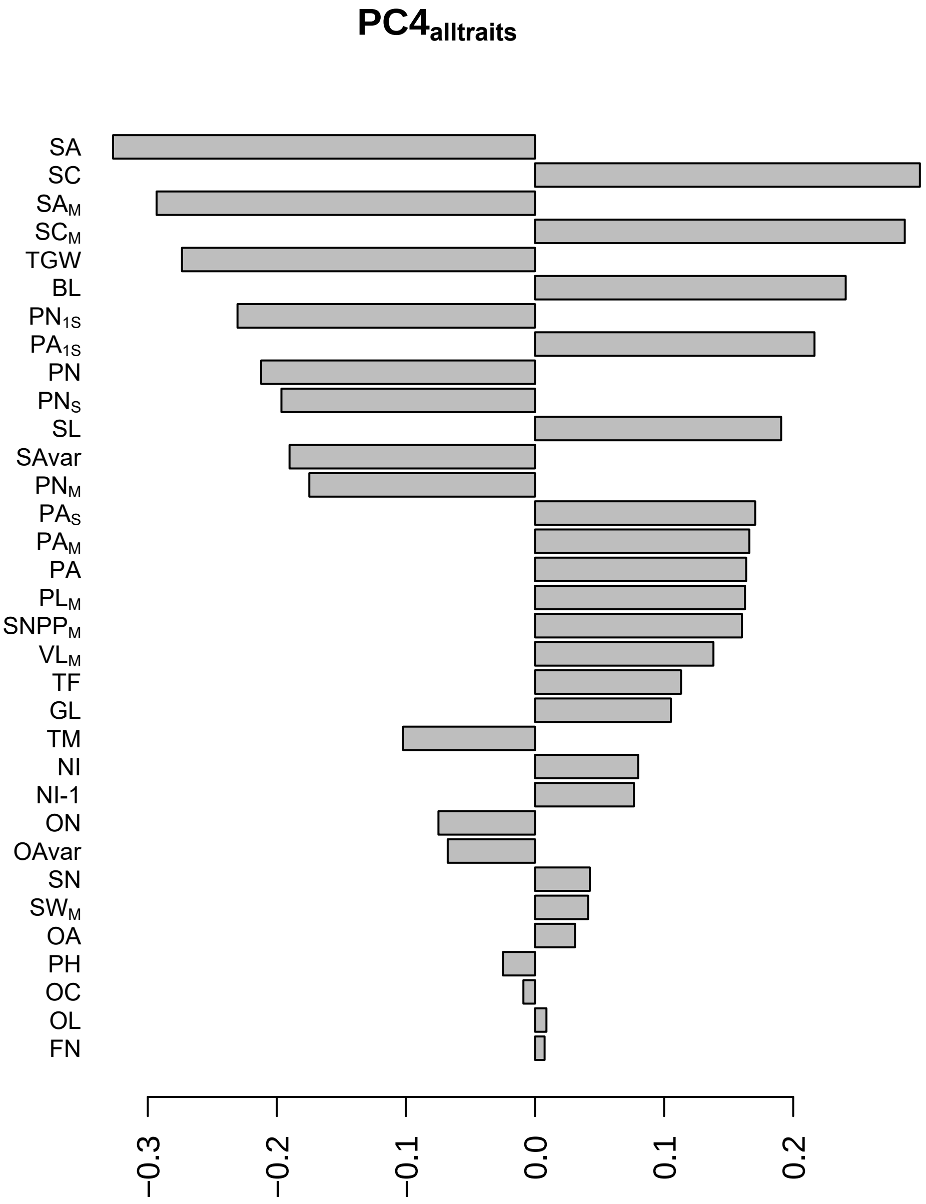

PC4alltraits
SA
SC
SAM
SCM
TGW
BL
PN1S
PA1S
PN
PNS
SL
SAvar
PNM
PAS
PAM
PA
PLM
SNPPM
VLM
TF
GL
TM
NI
NI-1
ON
OAvar
SN
SWM
OA
PH
OC
OL
FN
−0.3
−0.2
−0.1
0.0
0.1
0.2

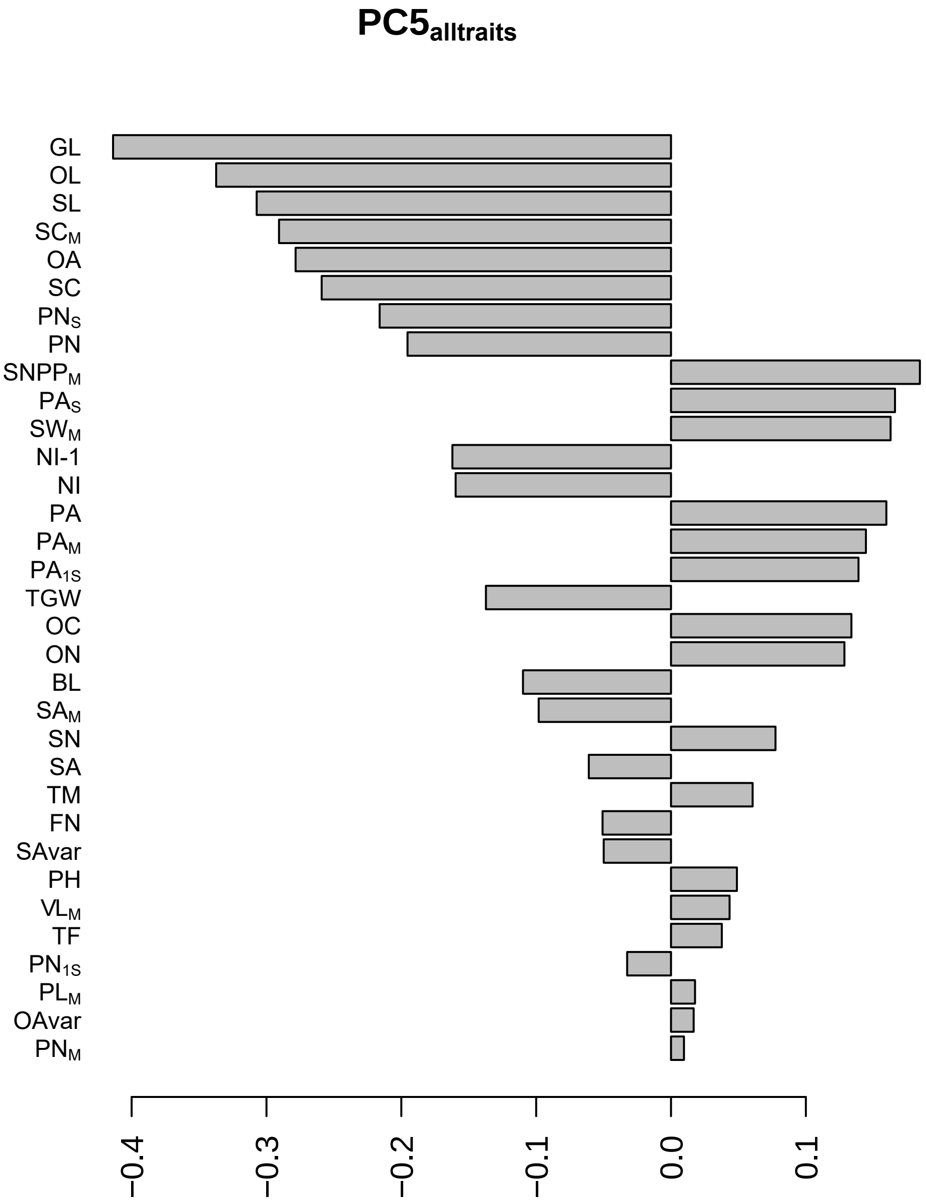

PC5alltraits
GL
OL
SL
SCM
OA
SC
PNS
PN
SNPPM
PAS
SWM
NI-1
NI
PA
PAM
PA1S
TGW
OC
ON
BL
SAM
SN
SA
TM
FN
SAvar
PH
VLM
TF
PN1S
PLM
OAvar
PNM
−0.4
−0.3
−0.2
−0.1
0.0
0.1

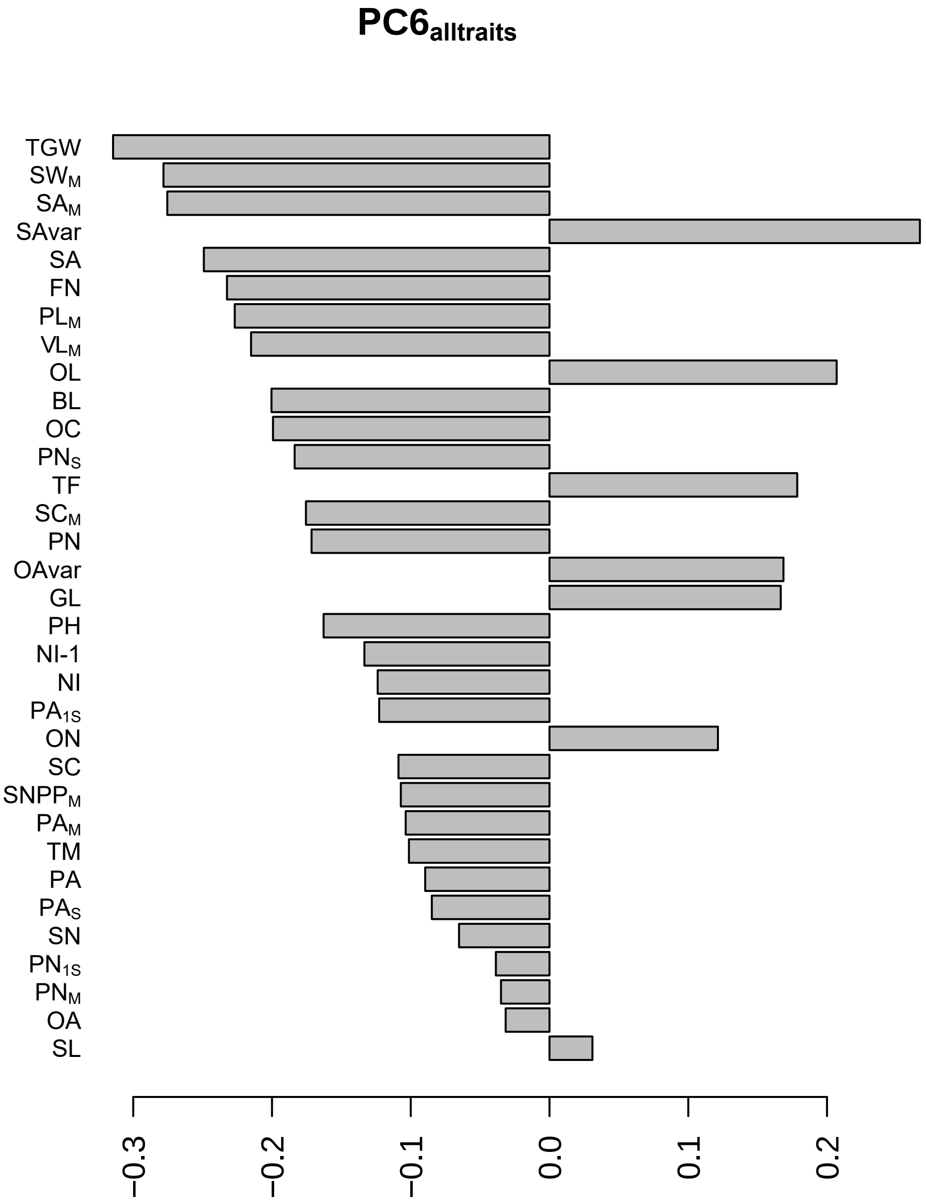

PC6alltraits
TGW
SWM
SAM
SAvar
SA
FN
PLM
VLM
OL
BL
OC
PNS
TF
SCM
PN
OAvar
GL
PH
NI-1
NI
PA1S
ON
SC
SNPPM
PAM
TM
PA
PAS
SN
PN1S
PNM
OA
SL
−0.3
−0.2
−0.1
0.0
0.1
0.2

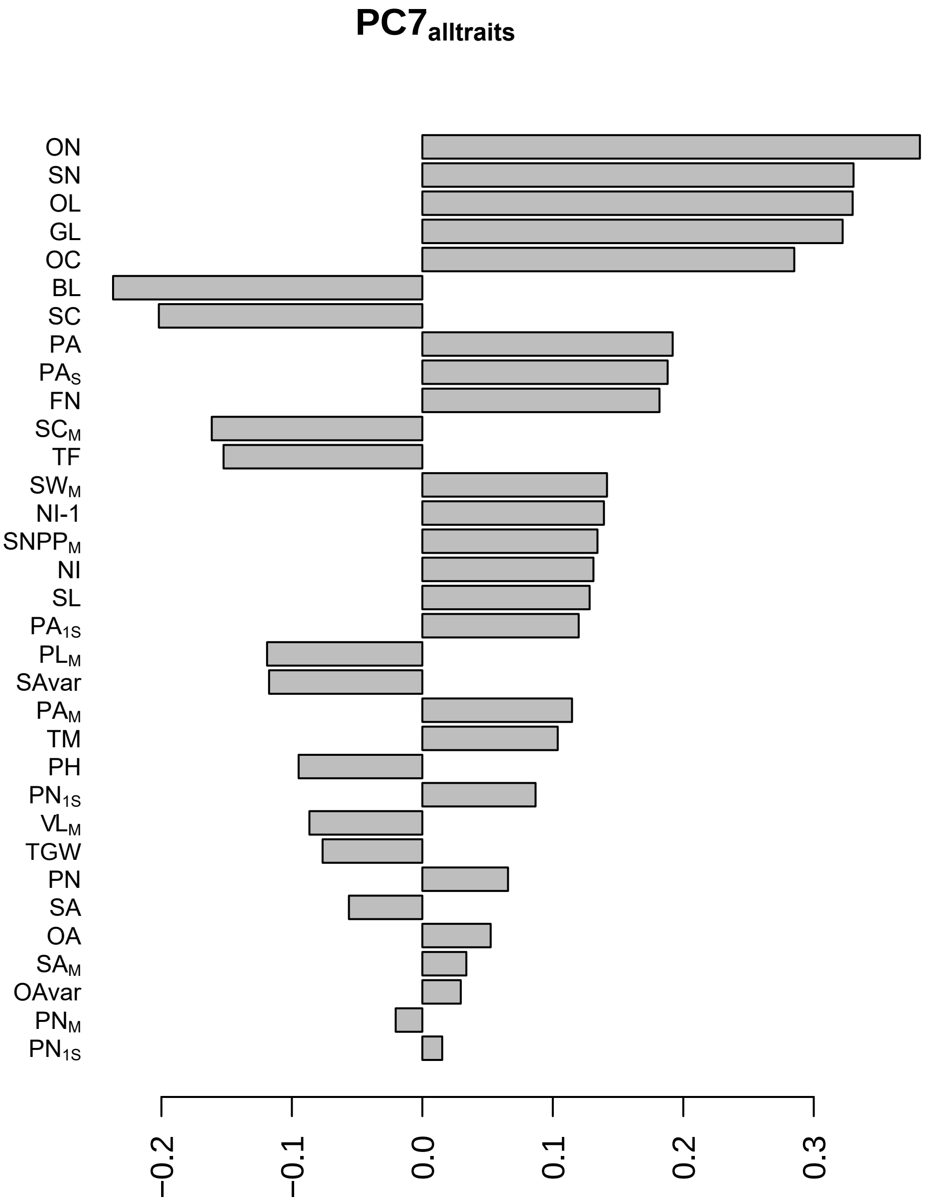
PC7alltraits
ON
SN
OL
GL
OC
BL
SC
PA
PAS
FN
SCM
TF
SWM
NI-1
SNPPM
NI
SL
PA1S
PLM
SAvar
PAM
TM
PH
PN1S
VLM
TGW
PN
SA
OA
SAM
OAvar
PNM
PN1S
−0.2
−0.1
0.0
0.1
0.2
0.3

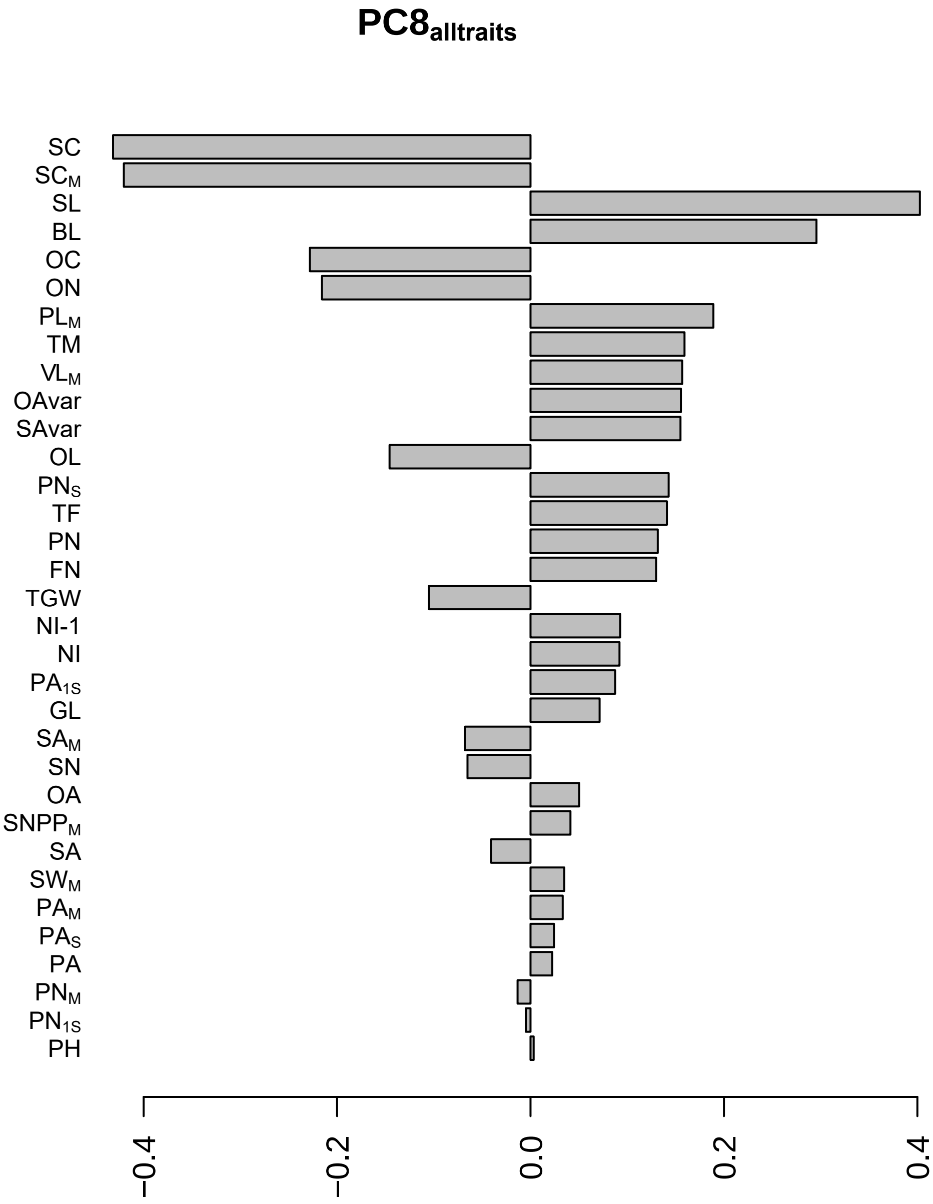
PC8alltraits
SC
SCM
SL
BL
OC
ON
PLM
TM
VLM
OAvar
SAvar
OL
PNS
TF
PN
FN
TGW
NI-1
NI
PA1S
GL
SAM
SN
OA
SNPPM
SA
SWM
PAM
PAS
PA
PNM
PN1S
PH
−0.4
−0.2
0.0
0.2
0.4

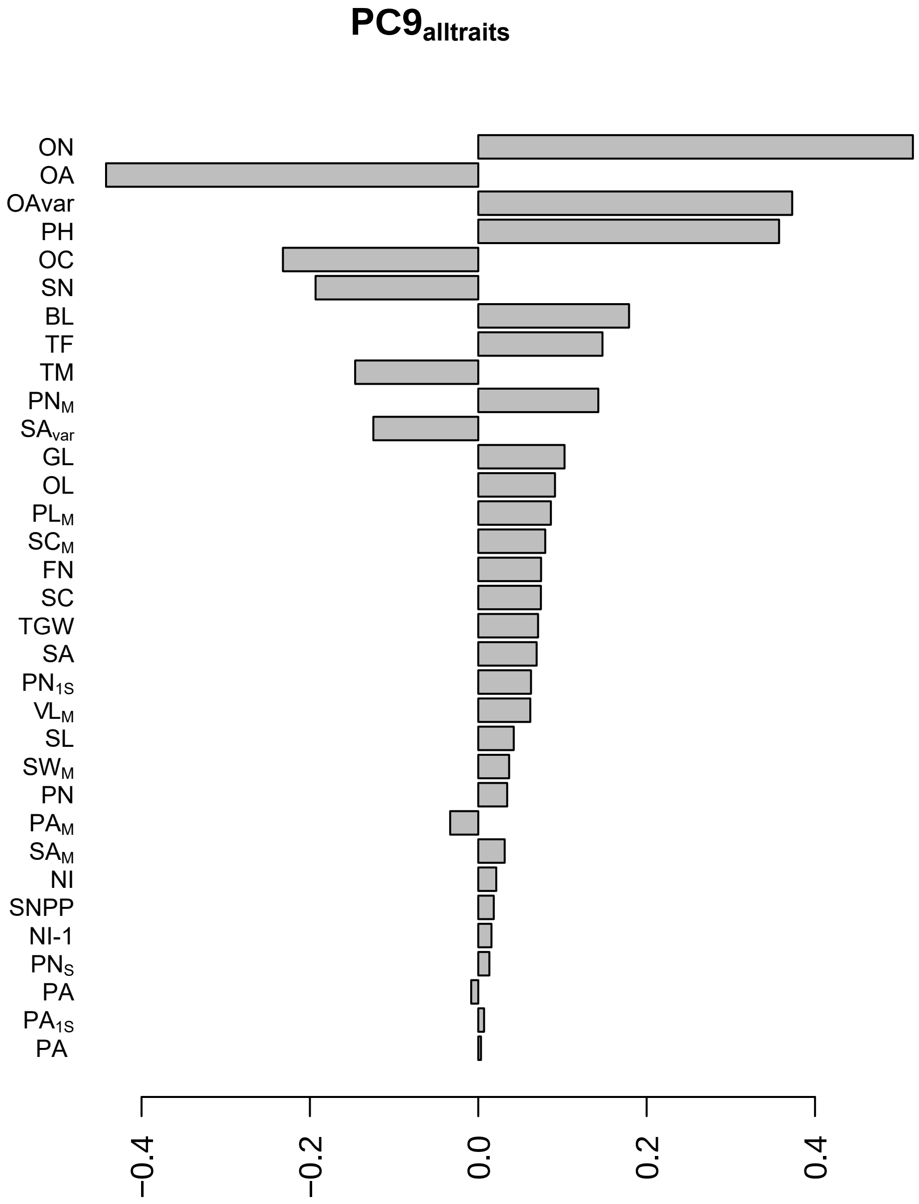
PC9alltraits
ON
OA
OAvar
PH
OC
SN
BL
TF
TM
PNM
SAvar
GL
OL
PLM
SCM
FN
SC
TGW
SA
PN1S
VLM
SL
SWM
PN
PAM
SAM
NI
SNPP
NI-1
PNS
PA
PA1S
PA
−0.4
−0.2
0.0
0.2
0.4

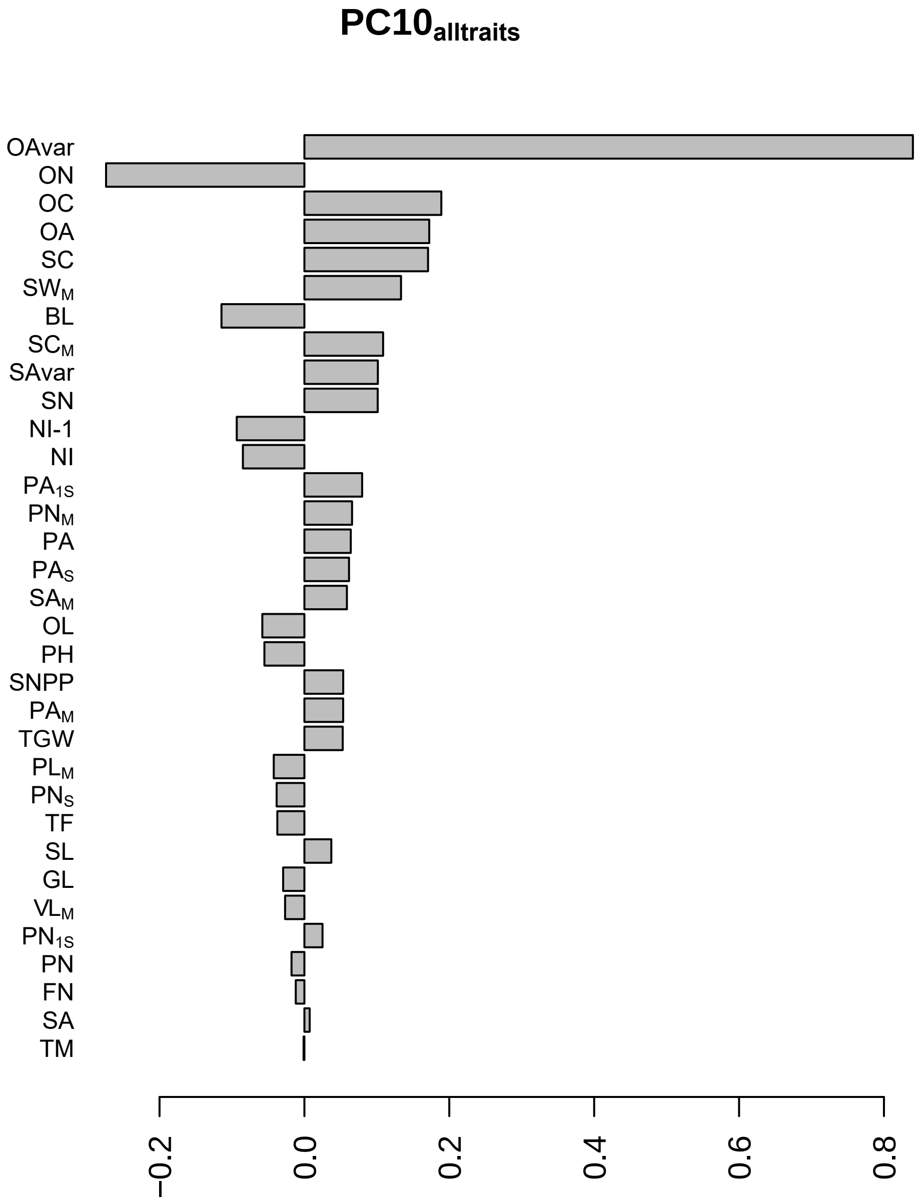
PC10alltraits
OAvar
ON
OC
OA
SC
SWM
BL
SCM
SAvar
SN
NI-1
NI
PA1S
PNM
PA
PAS
SAM
OL
PH
SNPP
PAM
TGW
PLM
PNS
TF
SL
GL
VLM
PN1S
PN
FN
SA
TM
−0.2
0.0
0.2
0.4
0.6
0.8

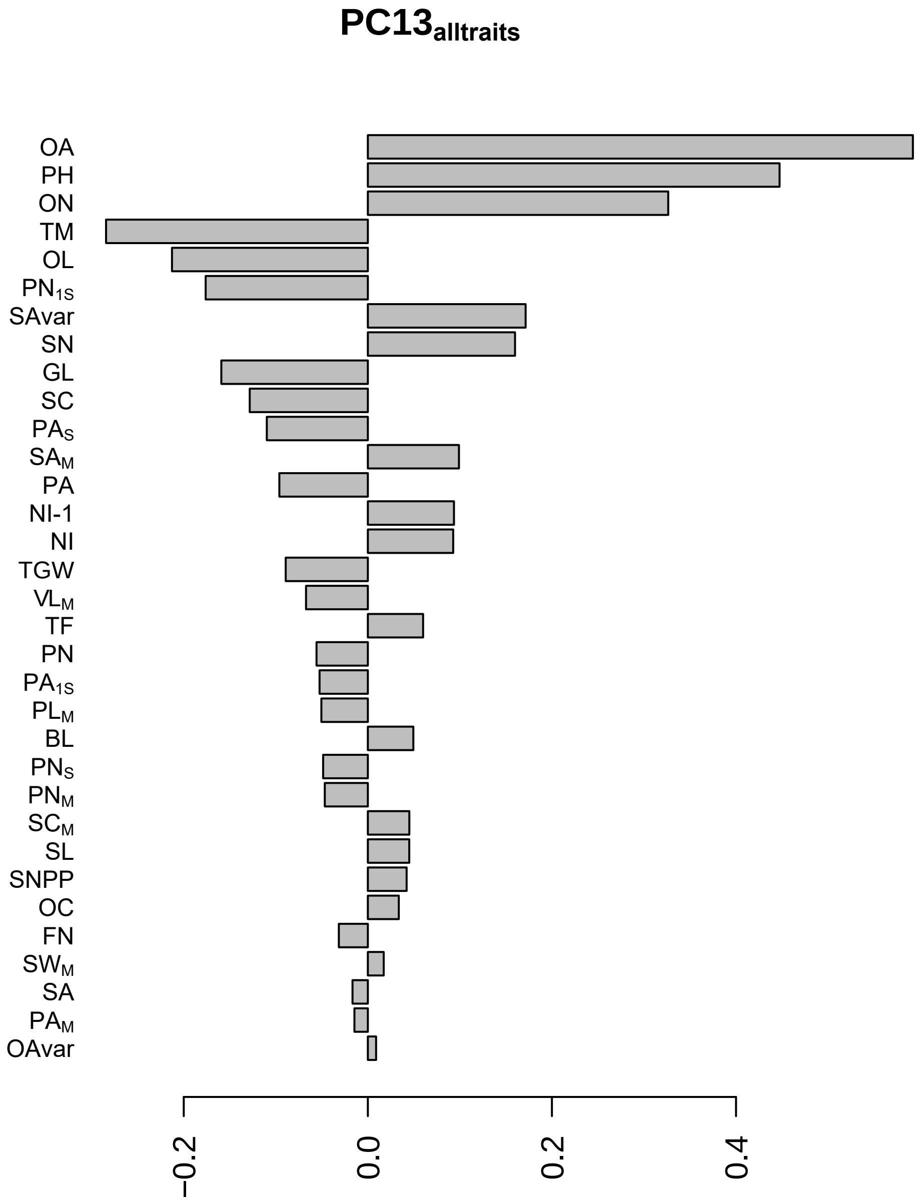
PC13alltraits
OA
PH
ON
TM
OL
PN1S
SAvar
SN
GL
SC
PAS
SAM
PA
NI-1
NI
TGW
VLM
TF
PN
PA1S
PLM
BL
PNS
PNM
SCM
SL
SNPP
OC
FN
SWM
SA
PAM
OAvar
−0.2
0.0
0.2
0.4

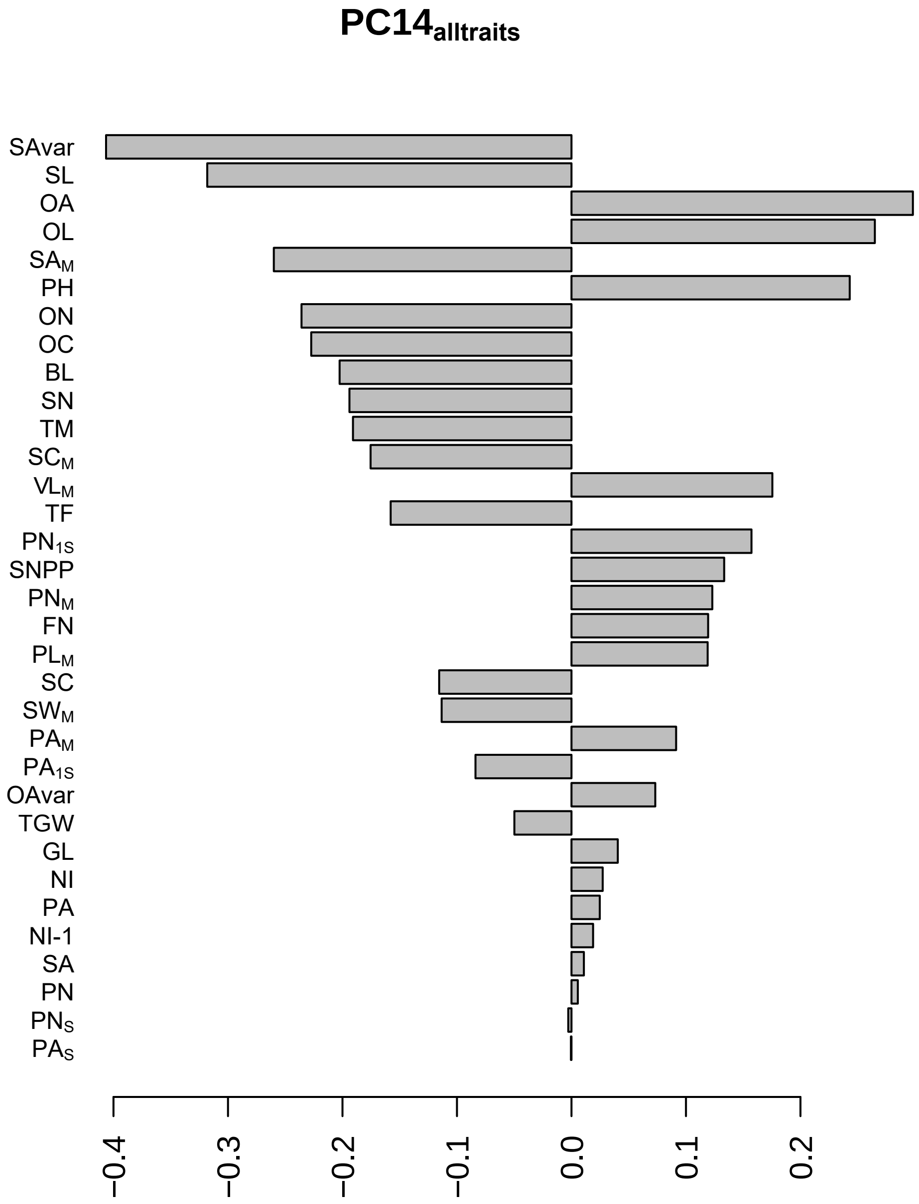

PC14alltraits
SAvar
SL
OA
OL
SAM
PH
ON
OC
BL
SN
TM
SCM
VLM
TF
PN1S
SNPP
PNM
FN
PLM
SC
SWM
PAM
PA1S
OAvar
TGW
GL
NI
PA
NI-1
SA
PN
PNS
PAS
−0.4
−0.3
−0.2
−0.1
0.0
0.1
0.2

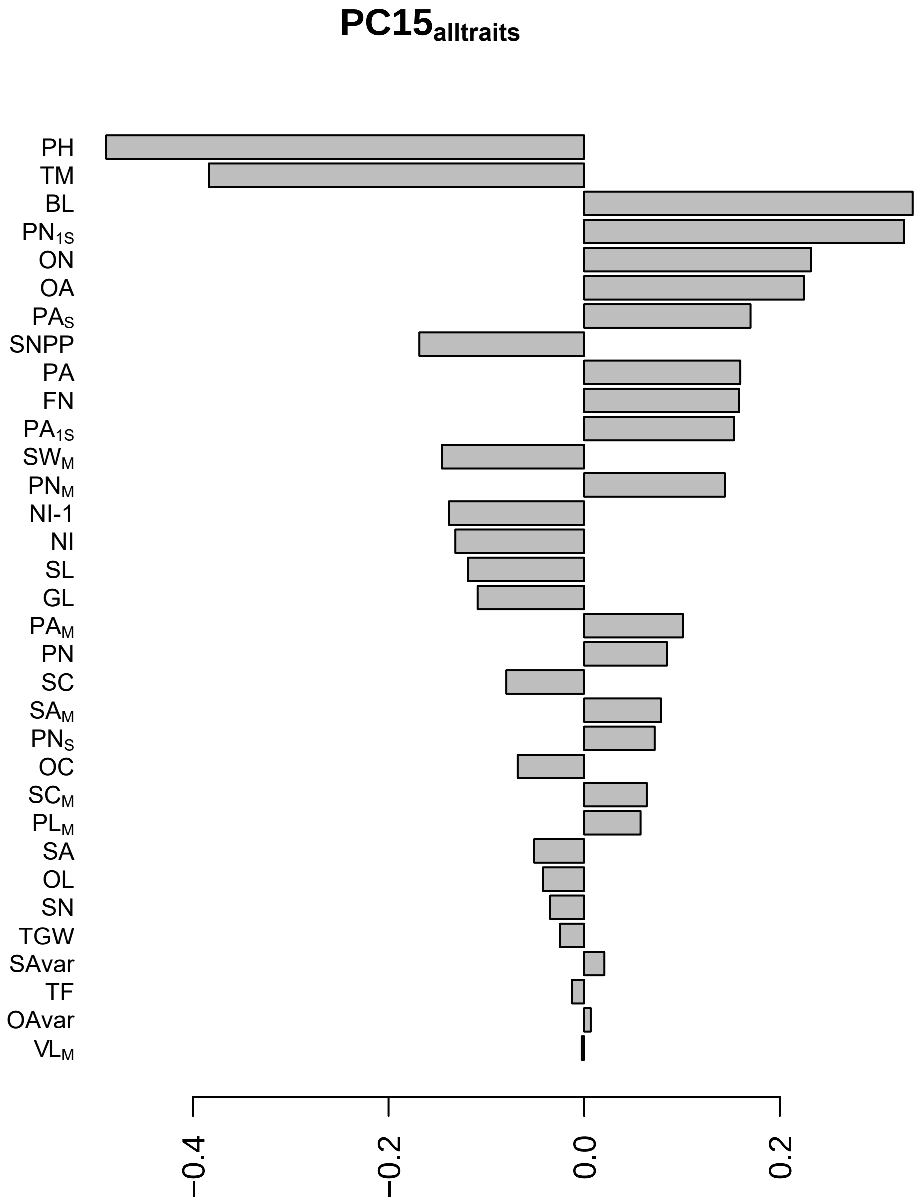
PC15alltraits
PH
TM
BL
PN1S
ON
OA
PAS
SNPP
PA
FN
PA1S
SWM
PNM
NI-1
NI
SL
GL
PAM
PN
SC
SAM
PNS
OC
SCM
PLM
SA
OL
SN
TGW
SAvar
TF
OAvar
VLM
−0.4
−0.2
0.0
0.2

Supplementary Table S1: List of 96 genotypes included in the diversity set population. The ASSYST code, genotype names, crop type description and the 4 oilseed rape groups are presented.

**ASSYST code**	**Genotype names**	**Crop type description**	**Four OSR groups**
BnASSYST-213	Abukuma Natane	Winter OSR	Winter OSR
BnASSYST-418	Alta Sweet	Swede	Other
BnASSYST-040	Apex	Modern winter OSR	Winter OSR
BnASSYST-090	Apex-93_5 x Ginyou_3 DH	Winter OSR	Winter OSR
BnASSYST-190	Aphid Resistant Rape	Winter fodder	Other
BnASSYST-133	Baltia	Winter OSR	Winter OSR
BnASSYST-091	Bienvenu DH4	Winter OSR	Winter OSR
BnASSYST-206	Brauner Schnitikohl	Siberian kale	Other
BnASSYST-273	Bronowski	Spring OSR	Spring OSR
BnASSYST-509	Cabernet	Winter OSR	Winter OSR
BnASSYST-510	Cabriolet	Winter OSR	Winter OSR
BnASSYST-185	Canard	Winter fodder	Other
BnASSYST-093	Canberra x Courage	Winter OSR	Winter OSR
BnASSYST-028	Capitol	Modern winter OSR	Winter OSR
BnASSYST-511	Castille	Winter OSR	Winter OSR
BnASSYST-512	Catana	Winter OSR	Winter OSR
BnASSYST-274	Ceska Krajova	Spring OSR	Spring OSR
BnASSYST-207	Chembere Dzagumhana	Unspecified	Other
BnASSYST-513	Chuanyou 2	Semiwinter OSR	Semiwinter OSR
BnASSYST-137	Coriander	Winter OSR	Winter OSR
BnASSYST-208	Couve Nabica	Cauve nabica	Other
BnASSYST-245	Cresor	Spring OSR	Spring OSR
BnASSYST-261	Cubs Root	Spring OSR	Spring OSR
BnASSYST-514	Dimension	Winter OSR	Winter OSR
BnASSYST-139	Dippes	Winter OSR	Winter OSR
BnASSYST-238	Drakkar	Spring OSR	Spring OSR
BnASSYST-275	Duplo	Spring OSR	Spring OSR
BnASSYST-193	Dwarf Essex	Winter fodder	Other
BnASSYST-194	English Giant	Winter fodder	Other
BnASSYST-263	Erglu	Spring OSR	Spring OSR
BnASSYST-101	Eurol	Winter OSR	Winter OSR
BnASSYST-515	Excalibur	Winter OSR	Winter OSR
BnASSYST-055	Expert	Modern winter OSR	Winter OSR
BnASSYST-516	Flash	Winter OSR	Winter OSR
BnASSYST-218	Groene Groninger Snijmoes	Unspecified	Other
BnASSYST-096	Hansen x Gaspard DH	Winter OSR	Winter OSR
BnASSYST-264	Helios	Spring OSR	Spring OSR
BnASSYST-410	Huguenot	Swede	Other
BnASSYST-517	Huron x Navajo	Winter OSR	Winter OSR

ASSYST code	Genotype names	Crop type description	Four OSR groups
BnASSYST-518	Inca x Contact	Winter OSR	Winter OSR
BnASSYST-107	Janetzkis Schlesischer	Winter OSR	Winter OSR
BnASSYST-411	Jaune A Collet Vert	Swede	Other
BnASSYST-251	Karat	Spring OSR	Spring OSR
BnASSYST-256	Karoo-057 DH	Spring OSR	Spring OSR
BnASSYST-150	Kromerska	Winter OSR	Winter OSR
BnASSYST-108	Lembkes Malchower (Lenora)	Winter OSR	Winter OSR
BnASSYST-102	Lesira	Winter OSR	Winter OSR
BnASSYST-105	Licrown x Express DH	Winter OSR	Winter OSR
BnASSYST-271	Liho	Spring fodder	Other
BnASSYST-097	Madrigal x Recital DH	Winter OSR	Winter OSR
BnASSYST-160	Matador	Winter OSR	Winter OSR
BnASSYST-268	Mazowiecki	Spring OSR	Spring OSR
BnASSYST-186	Moana, Moana Rape	Winter fodder	Other
BnASSYST-257	Monty-028 DH	Spring OSR	Spring OSR
BnASSYST-259	N02D-1952	Spring OSR	Spring OSR
BnASSYST-520	Ningyou 7	Semiwinter OSR	Semiwinter OSR
BnASSYST-258	N01D-1330	Spring OSR	Spring OSR
BnASSYST-109	Norin	Winter OSR	Winter OSR
BnASSYST-521	Palmedor	Winter OSR	Winter OSR
BnASSYST-522	POH 285, Bolko	Winter OSR	Winter OSR
BnASSYST-204	Q100	Synthetic	Other
BnASSYST-523	Quinta	Winter OSR	Winter OSR
BnASSYST-098	Rafal DH1	Winter OSR	Winter OSR
BnASSYST-209	Ragged Jack	Rape kale	Other
BnASSYST-168	Ramses	Winter OSR	Winter OSR
BnASSYST-221	Rapid Cycling Rape (CrGC5)	Rape kale	Other
BnASSYST-524	Rocket	Winter OSR	Winter OSR
BnASSYST-113	Samourai	Winter OSR	Winter OSR
BnASSYST-414	Sensation Nz	Swede	Other
BnASSYST-106	Shannon x Winner DH	Winter OSR	Winter OSR
BnASSYST-526	Shengliyoucai	Semiwinter OSR	Semiwinter OSR
BnASSYST-211	Siberische Boerenkool	Siberian kale	Other
BnASSYST-212	Slapska, Slapy	Unspecified	Other
BnASSYST-172	Slovenska Krajova	Winter OSR	Winter OSR
BnASSYST-239	Stellar DH	Spring OSR	Spring OSR
BnASSYST-260	Surpass400-024 DH	Spring OSR	Spring OSR
BnASSYST-229	SWU Chinese 1	Semiwinter OSR	Semiwinter OSR
BnASSYST-230	SWU Chinese 2	Semiwinter OSR	Semiwinter OSR
BnASSYST-203	Taisetsu	Winter vegetable	Other
BnASSYST-269	Tantal	Spring OSR	Spring OSR
BnASSYST-099	Tapidor DH	Winter OSR	Winter OSR
BnASSYST-527	Temple	Winter OSR	Winter OSR

ASSYST code	Genotype names	Crop type description	Four OSR groups
BnASSYST-436	Tina	Swede	Other
BnASSYST-283	Topas	Spring OSR	Spring OSR
BnASSYST-307	Tribune	Spring OSR	Spring OSR
BnASSYST-053	Verona	Modern winter OSR	Winter OSR
BnASSYST-401	Vige DH1	Swede	Other
BnASSYST-528	Vision	Winter OSR	Winter OSR
BnASSYST-270	Weihenstephaner	Spring OSR	Spring OSR
BnASSYST-240	Westar DH	Spring OSR	Spring OSR
BnASSYST-448	Wilhelmberger	Swede	Other
BnASSYST-394	Willi	Spring OSR	Spring OSR
BnASSYST-529	Xiangyou 15	Semiwinter OSR	Semiwinter OSR
BnASSYST-438	York	Swede	Other
BnASSYST-530	Zhongshuang II	Semiwinter OSR	Semiwinter OSR
BnASSYST-237	Zhouyou	Semiwinter OSR	Semiwinter OSR

Supplementary Table S2. List of transformations applied in order to satisfy homogeneity of variances.

Trait	Transformation
Plant height	square root
Number of flowering inflorescences	natural log
Number of secondary inflorescences	natural log
Number of flowers on the whole plant	natural log
Number of pods on secondary inflorescences	square root
Number of pods on the whole plant	square root
Pod abortion on the main inflorescence	logit with offset
Pod abortion in secondary inflorescences	logit with offset
Pod abortion in the whole plant	logit with offset
Ovary length	natural log
Time to maturity	natural log
Seed area from 10 pods from the main inflorescence	natural log
Seed compactness from 10 pods from the main inflorescence	logit
Seed area from the whole plant	natural log
Seed compactness from the whole plant	logit
Estimated total seed number from the whole plant (by TGW)	natural log
Seed oil content from the whole plant	logit, with reference of 50

Supplementary Table S3. Summary statistics over the whole population of plants. These include the mean, standard deviation (SD), the minimum (Min), maximum (Max), coefficient of variation (%CV) and heritability. All summaries have been calculated on the adjusted data backtransformed to the original scale. Heritability calculations have been done according to the formula, H^2=Var(Gene)/(Var(Gene)+Var(Env)), where Var(Gene) is the variance of the accession means and Var(Env) is the residual variance after fitting Trait ~ Line.

Trait	Mean	SD	Min	Max	CV (%)	Heritability
ON	33.42061	4.232212	21.09133	44.20224	12.66348	0.712146
OA	0.115021	0.01372	0.084351	0.172506	11.92822	0.708638
OL	4.85204	0.501377	3.123429	6.385621	10.33333	0.73875
GL	7.15939	0.654244	5.181047	9.601502	9.138268	0.668383
SL	2.059846	0.315774	1.10426	3.20125	15.32999	0.678755
BL	1.105276	0.297884	0.389241	2.615999	26.95113	0.725864
OAcvar	13.29828	3.016598	7.609162	28.03759	22.68412	0.340075
SY	13.83104	5.112593	-0.36286	30.23625	36.96463	0.691789
SW_M	0.660528	0.298372	-0.01849	1.795235	45.17171	0.559951
PN	293.8627	130.0789	0.168818	880.1546	44.26519	0.525376
PN_M	56.82148	22.56691	-3.10876	173.7208	39.71547	0.689118
PN_S	236.7674	121.5519	0.254323	827.3232	51.3381	0.499573
PL	6.009025	1.640704	1.651364	15.12342	27.30401	0.646169
PH	169.7785	27.88982	77.19692	288.8851	16.42719	0.73413
NI	9.868905	5.373592	1.997105	55.1263	54.44973	0.444512
NI-1	8.861261	5.369695	0.999851	53.90475	60.59741	0.446073
PA_M	28.64068	18.0737	1.621496	98.69986	63.10501	0.535236
PA_S	36.12051	17.59907	0.257428	99.86433	48.72319	0.566792
PA	34.62878	17.25387	0.22769	99.8836	49.82523	0.585134
FN	492.5712	277.9977	127.2368	2425.945	56.43808	0.51106
OC	40.97465	6.398335	6.915751	49.93014	15.61535	0.668214
SA_M	3.131293	0.572851	1.915669	4.979786	18.29438	0.81349
SC_M	0.900709	0.035014	0.767028	0.958851	3.887397	0.745762
SA	3.220794	0.578646	1.930691	5.160534	17.96594	0.80768
SC	0.908333	0.027741	0.803544	0.954271	3.054045	0.754221
PA_{1S}	36.24879	16.76044	-0.02158	100.2386	46.23723	0.572836
PN_{1S}	30.28568	12.65935	-0.55818	80.86194	41.7998	0.737021
TGW	4.669622	1.153336	2.128937	8.280727	24.69869	0.778309
SN	3187.737	1301.984	163.2147	6516.371	40.84353	0.713307
TF	102.8675	8.794261	87.99308	157.6276	8.549116	0.828945
TM	244.7853	14.77247	177.6758	270.7309	6.034871	0.746385
SAcvar	12.81114	3.075966	2.959941	30.21332	24.01009	0.556595
$SNPP_M$	14.48374	6.561572	0.332819	30.44099	45.30301	0.584962
VL	4.891266	1.432629	1.218655	12.67863	29.28953	0.640372

Supplementary Table S5. Ranks the Winter OSR genotypes according to its position within each PC_{macro} (n=5).

Genotypes	Seed yield (g)	$PC1_{macro}$	$PC5_{macro}$	$PC6_{macro}$	$PC7_{macro}$
POH 285, Bolko	21.29	18	24	30	34
Canberra x Courage	20.46	4	26	4	21
Norin	20.42	9	8	32	6
Shannon x Winner DH	20.28	25	1	31	8
Verona	20.21	11	7	7	26
Rafal DH1	19.98	7	9	1	38
Rocket	19.50	3	40	5	3
Madrigal x Recital DH	19.39	19	32	18	4
Capitol	19.04	26	12	8	22
Hansen x Gaspard DH	18.85	13	16	38	10
Inca x Contact	18.76	8	29	16	7
Expert	18.75	5	30	9	17
Huron x Navajo	18.41	1	27	14	13
Matador	18.32	2	10	2	11
Licrown x Express DH	18.21	33	28	6	27
Eurol	17.80	16	2	22	35
Vision	17.32	10	22	39	14
Temple	17.26	12	35	12	2
Dimension	17.06	23	19	11	29
Cabernet	16.75	21	39	17	9
Dippes	16.64	6	41	20	15
Coriander	16.41	22	38	29	28
Apex-93_5 x Ginyou_3 DH	16.25	28	20	36	25
Abukuma Natane	16.10	24	4	35	37
Palmedor	15.91	20	21	26	1
Apex	15.85	38	15	34	12
Lembkes Malchower (Lenora)	15.26	14	23	23	33
Baltia	15.09	31	3	40	32
Cabriolet	14.98	15	34	15	36
Tapidor DH	14.87	35	18	24	20
Kromerska	14.50	34	6	19	18
Slovenska Krajova	14.46	29	17	25	19
Castille	14.07	39	5	42	23
Ramses	13.70	17	37	28	30
Lesira	13.58	36	33	13	40
Excalibur	13.03	40	13	27	5
Quinta	13.02	30	25	41	41
Samourai	12.51	27	36	3	42
Catana	11.58	37	42	10	39
Janetzkis Schlesischer	11.34	32	31	21	31
Bienvenu DH4	10.80	41	14	37	24
Flash	5.38	42	11	33	16

Supplementary Table S6. Ranks the Spring OSR genotypes according to its position within each PC_{macro} (n=5).

Genotype	Seed yield (g)	$PC1_{macro}$	$PC5_{macro}$	$PC6_{macro}$	$PC7_{macro}$
Mazowiecki	15.99	1	1	14	19
Cresor	15.85	4	11	18	10
Tantal	15.83	10	8	7	8
Westar DH	14.99	5	14	6	2
Erglu	14.82	11	2	19	12
Ceska Krajova	14.70	6	15	11	1
N01D-1330	14.42	3	9	17	9
Topas	13.90	15	6	12	3
Willi	13.88	12	10	10	16
Duplo	13.48	7	18	20	4
Bronowski	13.43	8	17	8	6
Tribune	13.10	9	4	4	14
Drakkar	12.27	14	3	16	21
Helios	10.34	16	21	5	20
Karat	9.80	2	16	22	22
Monty-028 DH	9.59	13	22	1	17
N02D-1952	9.23	19	5	21	11
Karoo-057 DH	8.82	17	20	2	18
Surpass400-024 DH	7.67	18	13	13	13
Weihenstephaner	6.36	20	12	15	5
Stellar DH	4.36	21	19	9	15
Cubs Root	3.79	22	7	3	7

Supplementary Table S7: Ranks the Winter OSR genotypes according to its position within each $PC_{alltraits}$ (n=3).

Genotypes	Seed yield (g)	$PC1_{alltraits}$	$PC6_{alltraits}$	$PC7_{alltraits}$	$PC10_{alltraits}$
POH 285, Bolko	21.29	15	17	25	20
Canberra x Courage	20.46	12	20	18	18
Norin	20.42	17	1	41	8
Shannon x Winner DH	20.28	29	4	16	15
Verona	20.21	8	7	19	6
Rafal DH1	19.98	4	5	8	21
Rocket	19.50	1	42	2	7
Madrigal x Recital DH	19.39	22	28	23	14
Capitol	19.04	23	11	7	27
Hansen x Gaspard DH	18.85	11	38	14	5
Inca x Contact	18.76	7	29	12	29
Expert	18.75	10	30	22	4
Huron x Navajo	18.41	2	25	5	9
Matador	18.32	3	18	1	22
Licrown x Express DH	18.21	32	33	3	19
Eurol	17.80	16	2	21	25
Vision	17.32	13	36	30	2
Temple	17.26	14	32	10	30
Dimension	17.06	19	21	9	23
Cabernet	16.75	5	34	13	11
Dippes	16.64	9	27	39	33
Coriander	16.41	28	37	11	32
Apex-93_5 x Ginyou_3 DH	16.25	31	24	40	1
Abukuma Natane	16.10	21	14	33	13
Palmedor	15.91	24	10	24	16
Apex	15.85	37	16	36	3
Lembkes Malchower (Lenora)	15.26	18	22	17	26
Baltia	15.09	25	6	38	28
Cabriolet	14.98	6	31	26	41
Tapidor DH	14.87	34	41	6	35
Kromerska	14.50	26	3	37	10
Slovenska Krajova	14.46	33	15	32	17
Castille	14.07	38	9	27	31
Ramses	13.70	20	39	28	36
Lesira	13.58	39	19	35	12
Excalibur	13.03	41	23	4	34
Quinta	13.02	35	13	42	39
Samourai	12.51	27	35	20	42
Catana	11.58	36	40	34	24
Janetzkis Schlesischer	11.34	30	26	31	37
Bienvenu DH4	10.80	40	12	29	38
Flash	5.38	42	8	15	40

Supplementary Table S8. Ranks the Spring OSR genotypes according to its position within each $PC_{alltraits}$ (n=3).

**Genotype**	**Seed yield (g)**	**$PC1_{alltraits}$**	**$PC6_{alltraits}$**	**$PC7_{alltraits}$**	**$PC10_{alltraits}$**
Mazowiecki	15.99	1	2	21	8
Cresor	15.85	4	15	8	17
Tantal	15.83	9	1	20	16
Westar DH	14.99	6	14	6	13
Erglu	14.82	7	12	3	21
Ceska Krajova	14.70	11	17	9	2
N01D-1330	14.42	3	10	13	9
Topas	13.90	14	6	10	7
Willi	13.88	12	13	17	4
Duplo	13.48	8	19	5	6
Bronowski	13.43	5	21	1	15
Tribune	13.10	10	7	2	19
Drakkar	12.27	15	3	14	18
Helios	10.34	13	11	12	12
Karat	9.80	2	4	22	22
Monty-028 DH	9.59	16	22	11	20
N02D-1952	9.23	18	8	16	5
Karoo-057 DH	8.82	19	20	19	10
Surpass400-024 DH	7.67	17	5	7	3
Weihenstephaner	6.36	20	9	15	14
Stellar DH	4.36	21	16	18	11
Cubs Root	3.79	22	18	4	1

SupplementaryTable S9. Percentage of seed yield variation explained by different Partial Least Square (PLS) components for macrotraits for WOSR and SOSR.

Winter OSR		Spring OSR	
**PLS components**	**Seed yield variation (%)**	**PLS components**	**Seed yield variation (%)**
1	44.2	1	74.0
2	22.1	2	9.7
3	13.2	3	5.9
4	8.0	4	2.0
5	3.0	5	1.9
6	3.0	6	1.1
7	1.3	7	1.2
8	0.8	8	0.9
9	0.7	9	0.6

Supplementary Table S10: Percentage of seed yield variation explained by different Partial Least Square (PLS) components for alltraits for WOSR and SOSR.

Winter OSR		Spring OSR	
**PLS components**	**Seed yield variation (%)**	**PLS components**	**Seed yield variation (%)**
1	46.3	1	74.8
2	21.8	2	8.7
3	13.3	3	5.5
4	6.1	4	3.3
5	2.9	5	1.4
6	2.1	6	1.2
7	1.8	7	1.2
8	1.1	8	0.7
9	0.6		
10	0.6		
11	0.4		

Supplementary Table S11: Partial Least Squares (PLS) regression coefficients for Winter OSR and Spring OSR for macrotraits.

Trait	$WOSR_{macro}$	$SOSR_{macro}$
Estimated total seed number from the whole plant (by TGW)	1.2948	1.078
Thousand grain weight	0.5633	0.3728
Seed weight from 10 pods from the main inflorescence	0.1627	0.0763
Number of pods on the whole plant	0.1467	-0.0787
Number of pods on secondary inflorescences	0.1135	0.0106
Pod abortion in the whole plant	0.0453	-0.0854
Seed compactness from 10 pods from the main inflorescence	0.0433	-0.1274
Seed area from the whole plant	0.0426	0.0143
Seed compactness from the whole plant	0.0234	0.0655
Number of pods on the main inflorescence	0.0177	-0.0613
Time to flowering	0.0155	-0.0345
Pod abortion on the main inflorescence	0.0149	-0.0261
Time to maturity	0.0117	0.0146
Pod abortion on a secondary inflorescence	0.0086	0.0434
Valve length from 10 pods from the main inflorescence	-0.0023	-0.0053
Seed area from 10 pods from the main inflorescence	-0.0145	0.0809
Seed oil content from the whole plant	-0.0242	0.1415
Pod length from 10 pods from the main inflorescence	-0.0303	0.0186
Plant height (cm)	-0.0305	0.0464
Seed area coefficient of variation from whole plant	-0.0334	-0.005
Pod abortion in secondary inflorescences	-0.0405	-0.0079
Seed number/ pod from 10 pods from the main inflorescence	-0.0548	0.0412
Number of flowering inflorescences	-0.0799	0.0213
Number of flowers on the whole plant	-0.0808	0.0381
Number of secondary inflorescences	-0.087	0.0319
Number of pods on a secondary inflorescence	-0.1283	0.144

Supplementary Table S12: Partial Least Squares (PLS) regression coefficients for Winter OSR and Spring OSR for alltraits.

Trait	$WOSR_{alltraits}$	$SOSR_{alltraits}$
Estimated total seed number from the whole plant (by TGW)	1.2759	0.7794
Thousand grain weight	0.6184	0.1006
Seed weight from 10 pods from the main inflorescence	0.2216	0.1101
Number of pods on the whole plant	0.1976	-0.0229
Number of pods on secondary inflorescences	0.1642	0.0037
Pod abortion on a secondary inflorescence	0.1336	0.0971
Gynoecia length	0.0825	-0.008
Beak length	0.0521	-0.0388
Seed compactness from 10 pods from the main inflorescence	0.039	-0.1582
Plant height (cm)	0.0228	0.0459
Ovule area coefficient of variation	0.02	0.0028
Ovule area	0.0152	-0.003
Seed compactness from the whole plant	-0.0016	0.077
Seed area coefficient of variation from whole plant	-0.0047	0.0497
Seed oil content from the whole plant	-0.0049	0.342
Time to maturity	-0.0112	0.0734
Seed area from 10 pods from the main inflorescence	-0.013	0.1934
Time to flowering	-0.0134	-0.1001
Pod abortion on the main inflorescence	-0.0136	0.0679
Ovule number	-0.0343	0.0819
Pod abortion in the whole plant	-0.0511	-0.0827
Number of pods on the main inflorescence	-0.0513	0.087
Pod length from 10 pods from the main inflorescence	-0.0549	0.0639
Style length	-0.0581	0.014
Ovary length	-0.0616	-0.001
Valve length from 10 pods from the main inflorescence	-0.0709	0.0626
Pod abortion in secondary inflorescences	-0.0759	-0.0652
Seed area from the whole plant	-0.0867	-0.0064
Seed number/ pod from 10 pods from the main inflorescence	-0.0927	-0.0529
Number of flowering inflorescences	-0.0987	-0.0434
Number of secondary inflorescences	-0.1139	-0.0347
Number of flowers on the whole plant	-0.1325	0.1707
Number of pods on a secondary inflorescence	-0.1633	0.1171
